# Malaria Morbidity in Children in the Year after They Had Received Intermittent Preventive Treatment of Malaria in Mali: A Randomized Control Trial

**DOI:** 10.1371/journal.pone.0023390

**Published:** 2011-08-12

**Authors:** Alassane Dicko, Amadou Barry, Mohamed Dicko, Abdoulbaki I. Diallo, Intimbeye Tembine, Yahia Dicko, Niawanlou Dara, Youssoufa Sidibe, Gaoussou Santara, Toumani Conaré, Daniel Chandramohan, Simon Cousens, Paul J. Milligan, Diadier A. Diallo, Ogobara K. Doumbo, Brian Greenwood

**Affiliations:** 1 Malaria Research and Training Centre, Faculty of Medicine Pharmacy and Dentistry, University of Bamako, Bamako, Mali; 2 Centre de Santé de Référence de Kati, Kati, Mali; 3 Department of Infectious & Tropical Diseases, London School of Hygiene and Tropical Medicine, London, United Kingdom; Burnet Institute, Australia

## Abstract

**Background:**

Intermittent preventive treatment of malaria in children (IPTc) is a promising strategy for malaria control. A study conducted in Mali in 2008 showed that administration of three courses of IPTc with sulphadoxine-pyrimethamine (SP) and amodiaquine (AQ) at monthly intervals reduced clinical malaria, severe malaria and malaria infection by >80% in children under 5 years of age. Here we report the results of a follow-on study undertaken to establish whether children who had received IPTc would be at increased risk of malaria during the subsequent malaria transmission season.

**Methods:**

Morbidity from malaria and the prevalence of malaria parasitaemia and anaemia were measured in children who had previously received IPTc with SP and AQ using similar surveillance methods to those employed during the previous intervention period.

**Results:**

1396 of 1508 children (93%) who had previously received IPTc and 1406 of 1508 children (93%) who had previously received placebos were followed up during the high malaria transmission season of the year following the intervention. Incidence rates of clinical malaria during the post-intervention transmission season (July –November 2009) were 1.87 (95% CI 1.76 –1.99) and 1.73 (95% CI; 1.62–1.85) episodes per child year in the previous intervention and placebo groups respectively; incidence rate ratio (IRR) 1.09 (95% CI 0.99 –1.21) (P = 0.08). The prevalence of malaria infection was similar in the two groups, 7.4% versus 7.5%, prevalence ratio (PR) of 0.99 (95% CI 0.73–1.33) (P = 0.95). At the end of post-intervention malaria transmission season, the prevalence of anaemia, defined as a haemoglobin concentration<11g/dL, was similar in the two groups (56.2% versus 55.6%; PR = 1.01 [95% CI 0.91 – 1.12]) (P = 0.84).

**Conclusion:**

IPTc with SP+AQ was not associated with an increase in incidence of malaria episodes, prevalence of malaria infection or anaemia in the subsequent malaria transmission season.

**Trial Registration:**

ClinicalTrials.gov NCT00738946

## Introduction

Malaria is one of the most important infectious diseases in the world and 40% of the world's population is at risk. Despite increasing use of current control strategies such as rapid diagnosis and treatment of clinical cases, use of insecticide impregnated materials and indoor residual spraying with insecticides, malaria remains an important cause of morbidity and mortality, particularly in sub-Saharan Africa. More than 90% of the deaths due to malaria occur in this region and 88% of these deaths occur in children aged less than 5 years of age [1].

Intermittent preventive treatment of malaria (IPT), defined as the administration of a curative dose of an anti-malarial drug or drug combination at predefined time intervals to an at risk population regardless of whether or not they are known to be infected, is a promising new strategy for malaria control in areas where the infection still causes substantial morbidity and mortality [2]. Given to infants through the Expanded Programme of Immunization (EPI), IPT reduced the incidence of clinical malaria by 30% [3]. More marked reductions in the incidence of uncomplicated and severe malaria, in the range of 67% to 86%, have been obtained when children aged 0–5 years or 0–10 years were given IPT during the peak malaria transmission season [4]–[6]. The IPTc strategy is cost-effective [7] and a high level of coverage can be obtained when IPTc is delivered by community health workers [8].

The sample size of these initial studies was relatively small, with fewer than 500 subjects per treatment arm, and they were conducted in areas where the utilisation of insecticide treated bednets (ITNs) was low. Therefore, two larger studies of IPTc (about 1500 subjects per arm) were conducted in Mali and Burkina Faso in 2008 [9], [10] to determine the degree to which IPTc given during the malaria transmission season could reduce the incidence of clinical malaria in children who slept under an ITN. In the study conducted in Mali, children aged 3–59 months of age were randomized to receive IPT with sulphadoxine-pyrimethamine (SP) plus amodiaquine (AQ) or placebos on three occasions at monthly intervals during the 2008 malaria transmission season, starting in August 2008. All children received a long lasting insecticide treated net (LLIN) at the beginning of the malaria transmission season and were followed through the 2008 malaria transmission season (intervention period) to assess the safety and effects of the intervention. Results of the safety and efficacy of IPTc on the incidence of clinical malaria, severe malaria, malaria infection, anemia and anthropometric indicators during the 2008 malaria transmission season have been reported previously [9]. In brief, administration of IPTc resulted in an 82% reduction in the incidence of uncomplicated malaria and an 87% reduction in the incidence of severe malaria. The medication was well tolerated and there were no serious drug related adverse events.

A concern over the use of IPTc is that it may impair the acquisition of naturally acquired immunity leading to an increase in the incidence of malaria in the period after treatment is stopped, as was seen when chemoprophylaxis was given to African infants or children [11], [12]. No significant rebound in the incidence of clinical malaria in the year after IPTc was given was observed in the initial studies conducted in Senegal, Mali and Ghana [4]–[6]. However, a higher prevalence of anaemia at the end of the year following IPT administration was seen in the Senegalese study (10% in the IPT arm versus 6% in the placebo arm; p = 0,02) although there was no significant difference in mean haemoglobin concentrations between the two arms [13]. In Senegal, children who had received IPTc had lower malaria antibody titres than children who had received placebos [14]. For these reasons, we followed children recruited to the large IPTc trial in Mali to determine whether they were at increased risk of malaria in the year after IPTc was given.

## Methods

The protocol of the trial and its amendment (Protocol S1), and CONSORT checklist (Checklist S1) are available as supporting information.

Details of the study sites, the overall design of the trial and the surveillance procedures used have been published previously [9] and are, therefore, summarized only briefly in this paper. Similar surveillance procedures were used in the post-intervention follow-up period to those employed during the intervention period.

### Study sites

The study was conducted in two villages, Djoliba and Siby, and in the small town of Ouelessebougou in the district of Kati in the savannah region of Mali. Djoliba and Siby are located 40 and 30 km south west of the capital city Bamako respectively and Ouelessebougou is located 80 km south of Bamako. Each locality has a community health centre. A research team composed of physicians and medical residents was established in each of the three community health centres to follow up and provide health care to the study participants.

### Study design and participants

The study was an individually randomised, placebo controlled trial. A total of 3017 children aged 3–59 months were randomized in a 1: 1 ratio to receive IPT with sulphadoxine-pyrimethamine (SP) plus amodiaquine (AQ) (n = 1509) or their placebos (n = 1508) on three occasions at monthly intervals during the peak period of malaria transmission starting in August 2008. A long lasting insecticide treated net (LLIN) (PermaNet®; www.vestergaard-frandsen.com) was provided at enrollment to all the participants. Parents or guardians were instructed on how to use the net and the importance of using the net regularly was emphasized. Participants were followed to the end of the 2008 malaria transmission season (intervention period) to assess the safety and efficacy of the intervention and to the end of the 2009 transmission season to look for any rebound effect from the intervention given during the previous year.

### Endpoints

The primary endpoint for the follow-up study was the incidence of clinical malaria during the follow-up malaria transmission season (July to November 2009). Clinical malaria was defined as the presence of fever (axillary temperature ≥37.5°C) or a history of fever in the past 24 hours and the presence of *Plasmodium falciparum* asexual parasitaemia at a density greater or equal to 5,000 parasites per µl. Secondary endpoints were (i) the incidence of clinical malaria during the whole post-intervention period (December 2008 to November 2009), (ii) the incidence of severe malaria defined according to the WHO criteria [15], (iii) the prevalence of malaria infection defined as the presence of asexual *P. falciparum* parasitaemia during the high transmission season and at the end of the malaria transmission season, (iv) the prevalence of mild, moderate or severe anaemia defined as a haemoglobin (Hb) concentration<11 g/dL, <8 g/dL and <5 g/dL respectively at the end of the malaria transmission season and (v) the incidence of hospital admission defined as a stay of at least 24 hours in hospital for treatment during the follow-up period.

### Follow –up

Passive surveillance for clinical malaria continued after the end of the intervention period (6–7 weeks after the final round of IPTc had been given) to the end of the subsequent transmission season. Parents were encouraged to bring their child to a study health centre, where medical staff were available 24 hours a day and 7 days a week, if their child became unwell. A finger prick blood sample was obtained from any study child with fever (an axillary temperature of 37.5°C or higher) or a history of fever within the previous 24 hours for preparation of a blood film, measurement of Hb concentration and for a rapid diagnostic test (RDT) for malaria OPTIMAL IT® (Diamed AG, Cressier FR, Switzerland). Children who had a positive RDT for malaria were treated immediately with artemether/lumefantrine (AL). Severe cases were admitted to the health centre or referred to the paediatric ward of the Gabriel Touré Hospital in Bamako. Causes of death were assessed within a month of death using a modified version of the INDEPTH post-mortem questionnaire (http://www.indepth-network.org/index.php?option=com_ content&task  = view&id =  96&Itemid = 184).

Visits were made to the home of a randomly selected sample of 150 children each week during the post-intervention rainy season (July to November, 2009). During these home visits, the use of a LLIN was assessed by asking if the child had slept under an LLIN the previous night and the presence of the net was checked by field staff. In addition, the axillary temperature of each child was taken and a blood film obtained regardless of whether or not the child had fever to measure the prevalence of malaria infection.

At the end of the 2009 malaria transmission season, a cross-sectional survey of all the children enrolled into the study was carried out. A clinical examination was performed, thick and thin blood films were prepared and haemoglobin concentration was measured.

### Laboratory methods

Thick blood films were air dried, stained with Giemsa and examined for malaria parasites by two well-trained technicians. One hundred high power fields were examined before a film was declared negative. Parasite density was determined by counting the number of parasites present per white blood cell (WBC) on a thick smear and assuming a WBC count of 8,000 per µl. In the case of a discrepancy (positive/negative or a difference in parasite density greater than 30%), a third reading was done. The median parasite density of two or three readings was used. Haemoglobin concentrations were measured using a haemoglobin analyzer (Hemocue® HB 301, Angelholm, Sweden) on blood obtained by finger prick.

### Data management and analysis

Data were collected on standardized forms, double-entered and verified using MS Access and then exported to Stata (StataCorp, College Station, Texas, US) for additional cleaning and analysis. A data analysis plan was written and submitted to the DSMB prior to analysis. An intention-to-treat analysis was performed. The intervention period was defined as the period from the first dose of IPT administration to the end of November 2008 (42 days after the third dose of IPT). The post-intervention period of high malaria transmission was defined as the period from July to November 2009 and the whole post intervention period was defined as the period from December 2008 to November 2009, which included the low malaria transmission season (dry season from December 2008 to June 2009).

Incidence rates of clinical malaria, severe malaria and hospital admissions were calculated by dividing the number of episodes by the total child days at risk. Children were not considered at risk for 21 days after treatment for an episode of malaria and these days were not included in the calculation of the child days at risk. The incidence rates in the two treatment groups were compared using Cox regression to estimate the incidence rate ratio (IRR), with adjustment for age, gender and locality, and using a robust standard error to allow for the lack of independence among repeated episodes in the same child. Time to first episode of clinical malaria in the two arms was examined using Kaplan-Meier plots and compared using the log rank test. The prevalence of malaria infection was compared between the previous intervention and control groups using a generalized linear model, and prevalence ratios (PRs) were adjusted for age, sex and village.

Anthropometric data collected at the end of the 2009 malaria transmission season were analysed as previously described [9]. Z-scores for weight-for-age (WAZ; underweight), height-for-age (HAZ; stunting) and weight-for- height (WHZ; wasting) were determined. The WHO child growth standard (http://www.who.int/childgrowth/software/en/) was used to define wasting, stunting and being underweight and to code these variables as binary using z-scores with a score of<−2 as a cut-off [16]. A generalized linear model was fitted with age, sex and village as covariates to estimate relative risks (RRs) of anthropometric indicators for the comparison between children who had previously received IPTc and those who had received placebos during the previous year.

### Ethics

The study protocol was reviewed and approved by the Ethical Committee of the Faculty of Medicine, Pharmacy and Dentistry, University of Bamako, Mali and by the Ethics Committee of the London School of Hygiene and Tropical Medicine. Individual, written, informed consent was obtained from a parent or guardian of each child prior to screening and enrolment. A Data and Safety Monitoring Board (DSMB) was established and monitored the trial with the support of a local medical safety monitor. GCP monitoring of the trial was performed by PharmaClin (http://www.pharmaclin.com).

## Results

### Characteristics of children at the start of the follow-up high malaria transmission period

**Figure 1:**
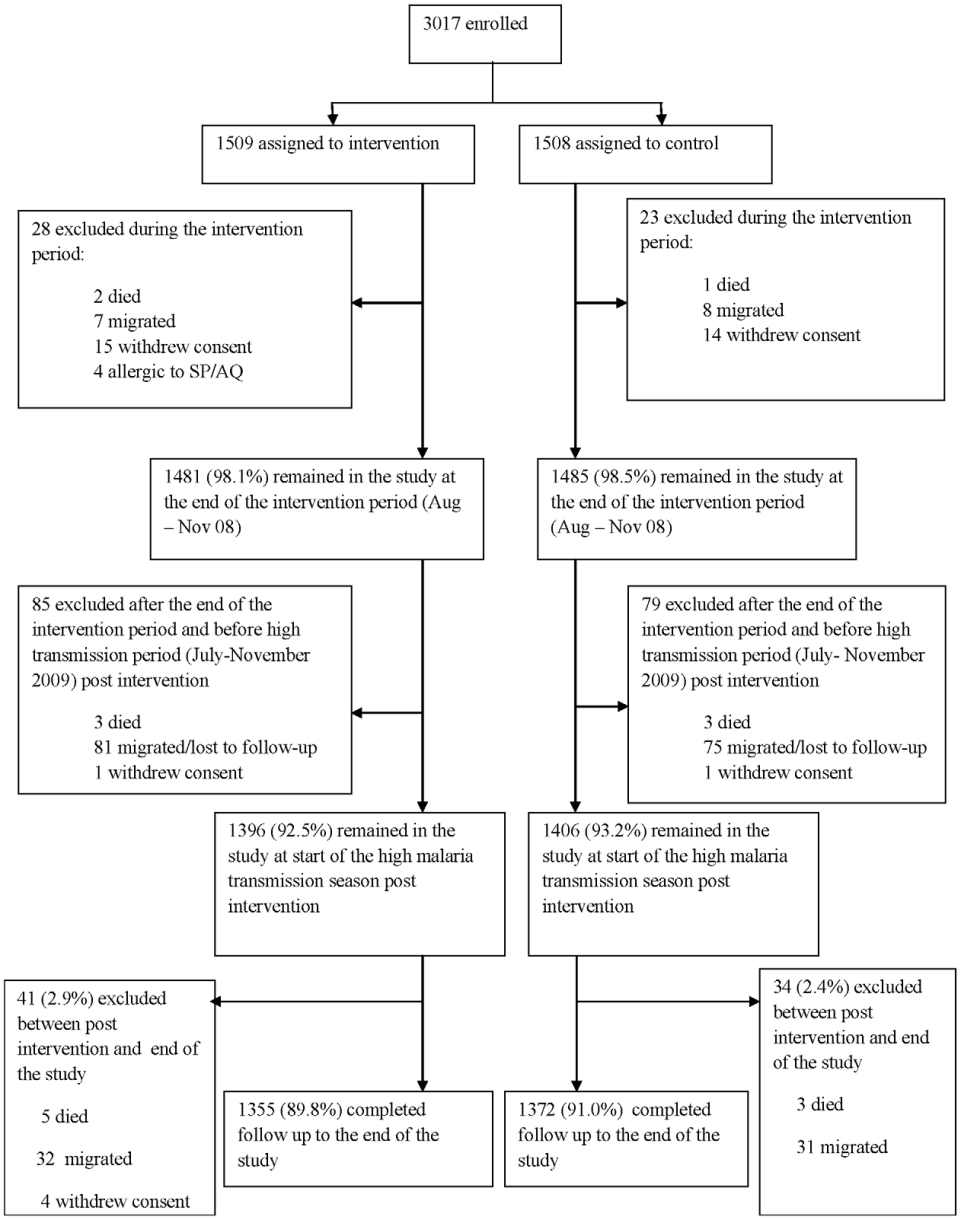
Study flow chart.

In 2008, 1509 and 1508 children were randomly assigned to receive IPTc with SP+AQ or placebos of these drugs respectively. One thousand three hundred and ninety-six of the 1509 children (92.5%) initially randomised to receive IPTc were available for follow-up on July 1^st^ 2009. Five children had died, the families of 16 withdrew consent, 4 children were allergic to SP or AQ and were therefore excluded, and 96 had migrated out of the study area (Figure 1). One thousand four hundred and six of the 1508 children (93.2%) assigned to receive placebos were available for follow-up on July 1^st^ 2009. Four of these children had died, the families of 15 withdrew consent and 83 had migrated out of the study area. During the high malaria transmission season, a further 41 subjects in the intervention arm were lost to follow-up (5 died, 32 migrated and 4 withdraw consent) and 34 were lost to the control arm (3 had died and 31 migrated). The proportion of children who completed the whole study was 89.8% (1355/1509) in the intervention arm and 90.9% (1372/1508) in the control arm (P = 0.29). The age and sex distributions of the children available for follow-up were similar in the two arms (Table 1). The mean age of the former intervention group was 40.6 months (95%CI 39.7–41.5) and that of the former control group 40.9 months (95%CI 40.0–41.7). The distribution of children between treatment arms was similar in the three study localities. The proportion of children seen during weekly visits who were reported to have slept under a LLIN during the previous night did not differ between children who had previously received IPTc (97.3%) or placebo (96.8%).

**Table 1:** Distribution of children by age, sex and village at the beginning of the high malaria transmission season in the post intervention year.

	Intervention	Control
	N = 1396% (n)	N = 1406% (n)
***Age (months)***		
12–23	21.5 (300)	20.2 (284)
24–35	21.6 (302)	21.7 (305)
36–47	19.9 (277)	20.4 (287)
> = 48	37.0 (577)	37.7 (530)
***Sex***		
Male	50.2 (706)	47.4 (662)
Female	49.8 (700)	52.6 (734)
***Locality***		
Djoliba	50.2 (281)	49.8 (279)
Ouelessebougou	50.1 (742)	49.9 (739)
Siby	50.7 (386)	49.3 (375)

### Incidence of clinical malaria during the post intervention period

The incidence of clinical malaria in the two study groups during the high malaria transmission period of the post intervention year is presented in Table 2. From July to November 2009, the high transmission period, 969 episodes of clinical malaria were recorded in the former intervention arm versus 913 in the former control group. The incidence of malaria in children from the former treatment arm was 1.87 (95%CI; 1.76–1.99) episodes per child year over the follow-up period compared with an incidence of malaria of 1.73 (95%CI 1.62 – 1.85) episodes per child year in those who had previously received placebos (IRR = 1.09; 95%CI 0.99–1.21) (P = 0.08). In children aged 24 months or more at enrolment there was a slightly greater tendency towards an increase in the incidence of malaria in children who had received IPTc (IRR = 1.12; 95%CI 0.99–1.26) (P = 0.06), but there was no evidence that the effect of IPTc on clinical malaria in the subsequent malaria transmission period was modified by age (P = 0.53). Analyses that considered the whole post intervention period (December 2008 to November 2009) (Table 3) showed a similar IRR when children who had received IPTc in 2008 were compared to children who had received placebos (IRR = 1.09; 95%CI 0.99–1.20) (P = 0.07).

**Table 2:** Incidence of malaria by age group during the post intervention, high malaria transmission season (July – November 2009).

	Former Intervention		Former Control					
Age † (months)	Episodes(child years)	Incidence rate$(95%CI)	Episodes(child years)	Incidence rate(95%CI)	UnadjustedIRR (95% CI)	P-value	Adjusted*IRR (95% CI)	P-value
<24	294 (212.0)	1.39 (1.24–1.55)	273 (205.3)	1.33 (1.18–1.50)	1.04 (0.86–1.26)	0.67	1.05 (0.86–1.26)	0.64
24+	675 (306.2)	2.20 (2.04–2.38)	640 (321.6)	1.98 (1.84–2.15)	1.12 (0.99–1.25)	0.07	1.12 (0.99–1.26)	0.06
Overall	969 (518.1)	1.87 (1.76–1.99)	913 (526.9)	1.73 (1.62–1.85)	1.08 (0.98–1.20)	0.12	1.09 (0.99–1.21)	0.08

† Age at the beginning of the intervention in August 2008.

$ Malaria episode was defined as fever or history of fever with 5000 or more asexual forms of *P. falciparum* per µL and absence of any other obvious cause of fever/illness.

*Adjusted for age sex, village and lack of independence among repeated episodes in the same child.

**Table 3:** Incidence of malaria by age group during the whole post-intervention period (December 2008 – November 2009).

	Former Intervention		Former Control					
Age† (months)	Episodes(child years)	Incidence rate$(95%CI)	Episodes(child years)	Incidence rate(95%CI)	UnadjustedIRR (95% CI)	P-value	Adjusted*IRR (95% CI)	P-value
< 24	335 (548.5)	0.61 (0.55–0.68)	315 (529.4)	0.59 (0.53–0.66)	1.02 (0.85–1.23)	0.80	1.03 (0.86–1.23)	0.77
24+	776 (803.2)	0.97 (0.90–1.04)	730 (833.8)	0.87 (0.81–0.94)	1.12 (0.99–1.25)	0.05	1.13 (1.00–1.27)	0.04
Overall	1111 (1352)	0.82 (0.77–.87)	1045 (1363)	0.77 (0.72–0.81)	1.08 (0.98–1.19)	0.12	1.09 (0.99–1.20)	0.07

† Age at the beginning of the intervention in August 2008.

$ Malaria episode was defined as fever or history of fever with 5000 or more asexual forms of *P. falciparum* per µL and absence of any other obvious cause of fever/illness.

*Adjusted for age sex and village.

**Figure 2:**
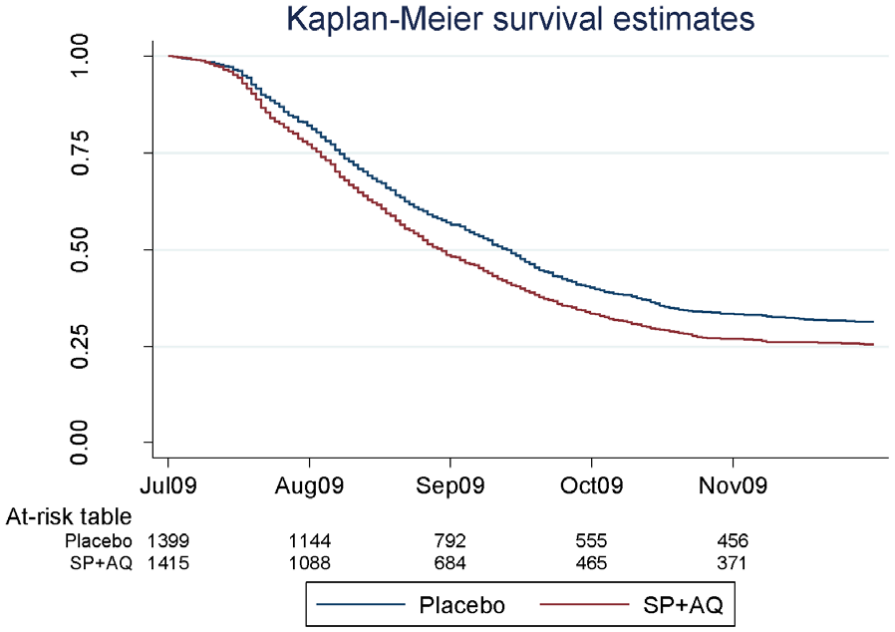
Survival estimates of the proportion of children who remained free of malaria, defined as fever or history of fever and a *P. falciparum* parasite density of at least 5000/µl, during the high malaria transmission season post intervention (July 2009 - November 2009).

Kaplan-Meier survival estimates of the proportion of children who remained free of malaria during the high malaria transmission period of the post intervention year were similar in both groups (Figure 2).

There was not strong evidence of an increase in the incidence of malaria post-intervention (data not shown) when a malaria episode was defined as fever or history of fever and positive asexual parasitaemia of any density (IRR = 1.07; 95%CI 0.98–1.18) (P = 0.13).

### Incidence of severe malaria during the post intervention period

The incidence of severe malaria in children in the two study groups during the post- intervention period is presented in Table 4. During the high transmission period of the post- intervention year, 16 cases of severe malaria occurred, 8 in the former intervention group (incidence rate = 0.0140 per child year at risk) and 8 in the former control arm (incidence rate = 0.0139 per child year) giving an IRR of 0.99 (95% CI 0.38–2.68) (P = 0.99). There were few additional cases in the intervening dry season, two in the group that had previously received IPTc and one in the group who had previously received placebos.

**Table 4:** Incidence of severe malaria in the high malaria transmission period of the post intervention year and during the whole post intervention period.

	Former Intervention (SP+AQ)		Former Control					
Periods	Episodes(child years)	Incidence rate(95%CI)	Episodes(child years)	Incidence rate(95%CI)	UnadjustedIRR (95% CI)	P-value	Adjusted*IRR (95% CI)	P-value
**Post intervention high malaria transmission season (Jul 09 – Nov 09)**	8 (570.7)	0.0140(0.0070–0.0280)	8 (575.9)	0.0139(0.0069–0.0278)	1.00(0.38–2.68)	0.99	1.00(0.38–2.68)	0.99
**Whole post-intervention period (Dec 08 – Nov 09)**	10 (1414.0)	0.0070(0.0038–0.0131)	9 (1411.0)	0.0064(0.0033–0.0122)	1.11(0.45–2.72)	0.81	1.11(0.45–2.74)	0.82

*Adjusted for age, sex and village.

### Incidence of hospital admissions and deaths post intervention and during the whole study period

During the high malaria transmission season of the post intervention period, eight hospital admissions were recorded in children who had previously received IPTc compared to seven in children who had received placebos. The incidence rates of hospital admission did not differ between the two groups of children (IRR = 1.16; 95%CI 0.42–3.23) (P = 0.77). This observation was not altered when the low malaria transmission (dry) season post intervention was included (IRR = 1.55; 95%CI 0.78–3.11) (P = 0.21) (Table 5).

**Table 5:** Incidence of all-cause hospital admissions in the high malaria transmission period of the post intervention year and during the whole post intervention period.

	Former Intervention (SP+AQ)		Former Control					
Periods	Episodes(child years)	Incidence rate(95%CI)	Episodes(child years)	Incidence rate(95%CI)	UnadjustedIRR (95% CI)	P-value	Adjusted*IRR (95% CI)	P-value
**Post intervention high malaria transmission season (Jul 09 – Nov 09)**	8 (570.7)	0.014(0.007–0.028)	7 (576.0)	0.012(0.006–0.025)	1.15(0.41–3.18)	0.78	1.16(0.42–3.23)	0.77
**Whole post-intervention period (Dec 08 – Nov 09)**	20 (1415)	0.014(0.009–0.022)	13 (1424)	0.009(0.005–0.016)	1.54(0.77–3.11)	0.22	1.55(0.78–3.11)	0.21

*Adjusted for age, sex and village.

During the high malaria transmission season of the post intervention year, five deaths were recorded amongst children who had previously received IPTc compared with four deaths among those who had previously received placebos.

### Malaria infection during the post intervention high transmission season

From July to November 2009, home visits were made to 1352 children who had previously received IPTc and to 1348 children who had previously received placebos to measure the prevalence of malaria infection; 172 and 168 children in the two groups were not found and microscopy results were available for 1179 and 1180 children respectively. The number of children who carried a malaria infection was 87 (7.4%) in children from the former intervention arm and 89 (7.5%) in children from the former control arm (PR = 0.99; 95%CI0.73–1.33) (P = 0.95). The geometric mean parasite densities in positive films were also similar in the former intervention (3360/µL; 95%CI 2017/µL–55401/µL) and control groups (3394/µL; 95%CI 1958/µL–5883/µL) (P = 0.96), the ratio between the two treatment arms was 1.01 (95%CI 0.48–2.12).

### Malaria infection, anaemia and anthropometric indicators at the end of the high malaria transmission season in the post intervention year

At the end of post intervention malaria transmission season, 1355 children from the former intervention group and 1372 children from the former control group were studied (Table 6). Microscopy slides for 7 and 16 children from these respective groups were lost or broken. Two hundred and twenty of the 1348 children (16.3%) in the former treatment arm were parasitaemic compared to 191 of the 1356 children (14.1%) in the former control group (PR = 1.17; 95%CI 0.96–1.14) (P = 0.12). Amongst children who had a malaria infection, the geometric means of parasite density were similar in the two treatment arms with a geometric mean of 3943 µ/L (95%CI 2863/µL–5431/µL) in the former intervention arm versus 4429 (95%CI 2979/µL–6002/µL) in the former control arm (P = 0.76).

**Table 6:** Malaria infection, anaemia, and anthropometric indicators in the post intervention period.

Outcomes	Former intervention		Former control		Unadjusted analysis		Adjusted analysis	
	% (n)	N	% (n)	N	Prevalence/Risk ratio(95%CI)	P-value	Prevalence/Risk ratio(95% CI)	P-value
**Weekly survey of malaria infection in the transmission season**								
Proportion with parasitaemia	7.4 (87)	1179	7.5 (89)	1180	0.98 (0.74–1.30)	0.88	0.99 (0.73–1.33)	0.95
(high malaria transmission season 2009)								
**End of transmission season survey**								
Proportion with parasitaemia	16.3 (220)	1348	14.1 (191)	1356	1.16 (0.97–1.39)	0.10	1.17 (0.96–1.14)	0.12
Proportion with anaemia (Hb<11 g/dl)	56.2 (759)	1350	55.6 (756)	1360	1.01 (0.94–1.08)	0.74	1.01 (0.91–1.12)	0.84
Proportion with moderate anaemia (Hb<8 g/dl)	2.9 (39)	1350	2.7 (37)	1360	1.06 (0.68–1.65)	0.79	1.05 (0.67–1.65)	0.84
Proportion with wasting†	4.7 (49)	1051	4.1 (43)	1057	1.15 (0.76–1.76)	0.49	1.14 (0.75–1.74)	0.54
Proportion with stunting$	20.8 (219)	1051	21.5 (227)	1057	0.96 (0.78–1.19)	0.72	0.98 (0.79–1.21)	0.85
Proportion with underweight£	11.3 (119)	1051	12.2 (129)	1057	0.92 (0.70–1.20)	0.53	0.94 (0.72–1.23)	0.66

† Wasting was defined as < −2 z score weight for age.

$ Stunting was define as < −2 z score of height for age.

£ Underweight was defined as < −2 z score of weight for height.

*Adjusted for age, sex and village.

During the 2009 end of malaria transmission season cross-sectional survey, haemoglobin concentrations were measured in 1350 children who had previously received IPTc and in 1360 children who had previously received placebos (Table 6). Mean Hb concentrations were similar in the two treatment arms (10.67 g/dL [95%CI: 10.60 g/dL–10.74 g/dL] versus 10.69 g/dL; [95%CI: 10.62 g/dL–10.76 g/dL]); the difference was only 0.019 g/dL (95%CI–008–0.118) (P = 0.70). The proportion of children who were anaemic (Hb<11 g/dL) was 56.2% in the former intervention arm and 55.6% in the former control arm (adjusted PR = 1.01; 95%CI 0.91–1.12) (P = 0.84). The proportions of children with moderately severe anaemia (Hb<8 g/dL) were similar in the two groups, 2.9% in children who had previously received IPTc and 2.7% in children who had previously received placebos (PR = 1.05; 95%CI 0.67–1.65) (P = 0.84).

Z scores were calculated for 1051 children from the former intervention arm and for 1057 children from the former control arm (Table 6). The proportions of wasted, stunted and underweight children amongst those who had previously received IPTc were 4.7% (49), 20.8% (219) and 11.3% (119) respectively. In children who had previously received placebos, the equivalent proportions were 4.1% (43), 21.5% (227) and 12.2% (129) respectively. There was no increased risk of wasting (RR = 1.14; 95%CI 0.75–1.74) (P = 0.54), stunting (RR = 0.98; 95%CI 0.79–1.21) (P = 0.85) or being underweight (RR = 0.94; 95%CI 0.72–1.23) (P = 0.66) post-intervention in children who had received IPTc during the previous high malaria transmission season.

## Discussion

This study assessed whether, in a context of a high usage of ITNs, children who had received intermittent preventive treatment with SP +AQ during one malaria transmission season were at increased risk of malaria morbidity during the subsequent malaria transmission season as consequence of lack of exposure to malaria parasites and consequent impairment of immunity. The study showed a small increase in clinical episodes of malaria during the post intervention high malaria transmission season in children who had previously received IPTc, a rate ratio of 1.09, with a confidence interval from 0.99 to 1.2. This is consistent with the results obtained in previous smaller studies undertaken in Mali, Senegal and Ghana that found no evidence of an important increase in malaria morbidity after administration of IPT in children for a single transmission season [4]–[6]. In the previous study in Senegal, there was a slightly increased risk of malaria among older children [4], which is also consistent with our findings, although in our study there was no evidence that the relative increase in risk varied with age (P = 0.53).

ITN usage in children enrolled in these three previous studies was low (<5%) while in our study more than 96% of the children used an ITN. A meta-analysis of the three previous studies found a similar increase (11%, 95% CI −1% to 24%) in malaria morbidity [13] suggesting that the use of ITN did not affect substantially the relative risk of malaria after the cessation of IPT in children. A surprising feature of this study was the high incidence of malaria in children who slept under an ITN during both the intervention and the follow-up years indicating that the use of an ITN alone is not sufficient to achieve a high degree of protection against malaria in this area of Mali. The efficacy of the nets and the absence of a high degree of resistance to pyrethroids in the study area was confirmed in 2009 [9].

In a parallel study conducted in Burkina Faso, with a similar design and a similar large sample size, a 12% increase in malaria morbidity was seen in the post intervention period, [Unpublished]. The confidence interval was narrower in that study, reflecting the larger number of malaria episodes.

In line with previous studies [13], as well as with the parallel study in Burkina Faso [Unpublished], there was no increase in severe morbidity (severe malaria, hospital admission or death) during the follow-up period. However, despite its relatively large size, our study had limited power to detect differences in these end-points. Nevertheless, the fact that no study has so far suggested an increase in severe disease is reassuring.

No evidence was found for an increase in malaria parasite prevalence or parasite density in subjects who had previously received IPTc during the post-intervention period in line with the results of previous studies [4], [6], [13]. In the parallel study in Burkina Faso, the proportion of children with malaria parasite at weekly visits during the post-intervention period was lower in subjects who had received IPT in the previous malaria transmission season but similar between the two groups at the end of the subsequent transmission season. However, mean parasite density was higher during the subsequent transmission season in children who had previously received IPTc in the Burkina Faso study [Unpublished].

At the end of the post intervention malaria transmission season, the prevalence of anemia, moderate anemia and nutritional indicators (wasting, stunting and being underweight) were similar among the subjects who had previously received IPTc and those who had not. Similar results were found in the parallel study in Burkina Faso [Unpublished]. However, Kweku et al [6] found a small increase in anemia in the intervention group in the year following the intervention period.

In summary, the results of this study are internally consistent, and consistent with those of other similar studies in different settings with different levels of malaria transmission, in showing that administration of an effective IPTc regimen for one malaria transmission season may result in a small increase in the incidence of clinical episodes of malaria during the subsequent malaria transmission season but no increase in the prevalence of malaria parasitaemia, anaemia or malnutrition. It is possible that the risk of rebound malaria may be influenced by the level of transmission but currently there are too few data to determine this. No increase in severe malaria has been seen in this or any other study but the numbers of subjects studied so far have been too small to exclude a small effect. The possibility of a small rebound effect must be set against the major benefit seen during the period in which the intervention was given, with an 80% reduction in the incidence of severe malaria and a probable, substantial reduction in all cause mortality [13].

A limitation of this and previous studies that have assessed the post-intervention consequences of IPTc is that the interventions have been given during a single malaria transmission season whilst, if IPTc is to be deployed as a malaria control tool, it is envisaged that children would receive seasonal IPTc for several years and it is possible that this would have a bigger impact on the acquisition of naturally acquired immunity than seen in this study. In a previous study of seasonal chemoprophylaxis undertaken in an area of the Gambia with a pattern of malaria similar to that of Mali, the risk of a rebound in clinical malaria increased with each year that a child had received seasonal prophylaxis only becoming substantial when a child had received prophylaxis for several seasons following birth [11]. There was no increase in mortality in the Gambian study. However, the findings of this Gambian study indicate that if IPTc is recommended as policy and implemented as part of national malaria control programmes in African countries with highly seasonal transmission of malaria, long-term follow-up of the impact of IPTc on naturally acquired immunity will be essential.

## Supporting Information

Protocol S1 Trial Protocol
(DOC)

Checklist S1 CONSORT checklist
(DOC)
