# Supplementary material for: Malaria Morbidity in Children in the Year after They Had Received Intermittent Preventive Treatment of Malaria in Mali: A Randomized Control Trial
Source: PLoS One. 2011 Aug 12;6(8):e23390. doi: 10.1371/journal.pone.0023390 (PMC3155530; doi:10.1371/journal.pone.0023390)
Supplement: Protocol S1 — Trial Protocol (DOC) [file pone.0023390.s001.doc]

**Amended Trial Protocol - June 2008**

# **I. Proposal Information**

A. Organization

| **Organization Name:** | London School of Hygiene & Tropical Medicine | |
| --- | --- | --- |
| **U.S. Tax Status (Refer to** [**Tax Status Definitions**](http://www.gatesfoundation.org/GlobalHealth/Grantseekers/HowToApply/RelatedInfo/TaxStatusDefinitions.htm)**)[[1]](#footnote-2):** | | Foreign Government |

Institutional Official authorized to submit and accept grants on behalf of organization:

| Prefix | Ms | First name | Penny | | Surname | Ireland | | *Suffix* |  |
| --- | --- | --- | --- | --- | --- | --- | --- | --- | --- |
| Title |  | | | Telephone | | | + 44 207 299 2678 | | |
| Address | London School of Hygiene &Tropical Medicine, Keppel St., London WC1E 7HT, UK | | | Fax | | | + 44 207 580 5636 | | |
|  | | | | | |
| E-mail | penny.ireland@lshtm.ac.uk | | | | | | | | |
| Web site | www.lshtm.ac.uk | | | | | | | | |

**B. Project**

| **Project Name:** | A trial of the combined impact of intermittent preventive treatment and insecticide treated bednets on morbidity from malaria in African children. |
| --- | --- |

Principal Investigator/Project Director:

| Prefix | Prof | First name | Brian | | Surname | Greenwood | | *Suffix* |  |
| --- | --- | --- | --- | --- | --- | --- | --- | --- | --- |
| Title | Professor of Tropical Medicine | | | Telephone | | | + 44 207 299 4707 | | |
| Address | London School of Hygiene &Tropical Medicine, Keppel St., London WC1E 7HT, UK | | | Fax | | | + 44 207 299 4720 | | |
|  | | | | | |
| E-mail | brian.greenwood@lshtm.ac.uk | | | | | | | | |
| Web site | www.lshtm.ac.uk | | | | | | | | |

| **Amount Requested From Foundation ($USD):** | $ 2.99M | **Project Duration (months):** | 24 months |
| --- | --- | --- | --- |
| **Estimated Total Cost of Project ($USD):** | $ 3.10M |  |  |
| **Organization’s total revenue for most recent audited financial year ($USD):** | $ 111 million |  |  |

***Charitable Purpose:***

| To demonstrate that intermittent preventive treatment with antimalarials adds to the protection against malaria provided by insecticide treated bednets by means of a controlled trial conducted in Burkina Faso, Ghana and Mali. |
| --- |

**Project Description:**

| The funds requested will be used to support a trial of intermittent preventive treatment in Burkina Faso, Ghana and Mali. Some capital expenditure will be required at each field site. This will comprise funds for a 4 x 4 vehicle for each site, motorcycles and some simple laboratory equipment. Salary costs at each site will include those needed for the project physicians, field staff and laboratory staff. The project will be coordinated by the LSHTM and a research fellow will be appointed to undertake this coordinating role. Consumables costs at each site will include those needed for the provision of ITNs for each trial participant, the drugs needed for IPT and for a contribution to the costs of routine patient care for study children. Local travel costs will be high as a substantial amount of field work will be required. Funds will be required for the investigators meeting needed to ensure standardization of methods across sites. |
| --- |

**C. Regulatory Approval Questionnaire**

Please enter an “x” in the appropriate column.

|  | Yes | No |
| --- | --- | --- |
| 1. Project will involve collaboration with for-profit companies |  | X |
| 2. Project will involve use or creation of intellectual property |  | X |
| 3. Proposal contains proprietary information |  | X |
| If you marked “yes” for any of the above statements, please complete Section VI.B. | | |
| 4. Project will involve research using vertebrate animals |  | X |
| 5. Project will involve research using human subjects | X |  |
| 6. Project will involve clinical trials | X |  |
| 7. Project will involve use of any of the following substances: • recombinant DNA subject to regulation • pathogens/toxins identified as “select agents” by U.S. law:  (http://www.aphis.usda.gov/vs/ncie/pdf/agent_toxin_list.pdf) • biohazards or genetically modified organisms |  | X |

If you marked “yes” for any of the above statements, please ensure that assurances are discussed in Section IV. Please refer to the additional guidelines in Section XI.A for more information on this topic.

# **II. Executive Summary**

- Goals and Objectives

The goal of this project is to determine the degree to which malaria can be prevented in children who sleep under an insecticide treated bednet (ITN) by the administration of intermittent preventive treatment (IPT) with an anti-malarial drug during the malaria season in countries where the transmission of malaria is seasonal.

The objective of the project is to conduct a randomized, placebo-controlled trial of IPT in children who sleep under an ITN at three sites in Africa where malaria transmission is highly seasonal. This trial will determine whether IPT adds to the protection provided by ITNs and whether the combined use of these interventions impairs the development of naturally acquired immunity to malaria, as reflected by an increase in the incidence of malaria in the year after children have received IPT.

Although a small number of trials of IPT in older children have been done, these have been conducted against a background of variable and uncontrolled use of bednets. No randomized trial has formally addressed the value of IPT in children who use an ITN. The outcome of the trial proposed will define the added value of IPT in ITN users and help to guide policy on the use of IPT in areas with seasonal malaria transmission where nets are used widely.

- Background and Rationale

Malaria remains a major cause of mortality and morbidity among young children in Africa and there are few effective tools available to prevent it. Vector control, using ITNs or curtains or indoor residual spraying (IRS), can reduce mortality and morbidity from malaria substantially [1] but, in most epidemiological situations, these interventions provide only imperfect protection and additional control measures are needed.

Intermittent preventive treatment in infants (IPTi) is a promising new approach to the prevention of malaria in infants. In Tanzania, administration of a treatment dose of sulfadoxine/pyrimethamine (SP) to infants at the time of second and third DPT and measles vaccinations led to a 50% reduction in the incidence of clinical attacks of malaria and anemia [2]. Surprisingly, protection persisted into the second year of life [3]. Similar, impressive results were obtained in a second Tanzanian study in which amodiaquine was given at growth monitoring clinics during the first year of life [4]. However, in Ghana and southern Mozambique less dramatic, although still significant, levels of protection against clinical attacks of malaria have been seen but without persistence of protection beyond infancy [5, 6]. Further trials of IPTi with SP have been completed in Ghana and in Kenya and it is expected that by the end of 2006, sufficient information will have been obtained to allow an informed recommendation to be made as to whether IPTi should be implemented routinely in areas with a high level of malaria transmission.

Results obtained with IPTi are encouraging but in many parts of Africa, the main burden of malaria is not in the first year of life but in older children. This is illustrated by the age pattern of malaria among nearly 20,000 cases of severe malaria collected at five sites in Africa through the SMAC network [7]. Only about 25% of these children were under the age of one year. In parts of Africa, such as much of the Sahel and sub-Sahel where malaria transmission is very seasonal, the peak age incidence for severe malaria is two to three years of age. For this reason, trials have been undertaken in areas of seasonal malaria transmission to determine whether IPT can be used as an effective malaria control tool in older children as well as in infants. In Mali, a 40% reduction in the incidence of clinical attacks of malaria was seen when two doses of SP were given eight weeks apart during the malaria transmission season [8]. More dramatic results were obtained in Senegal where administration of SP plus a single dose of artesunate (AS) on three occasions at monthly intervals during the peak malaria season reduced the incidence of clinical attacks of malaria by 86% [9]. IPT with SP and amodiaquine (AQ) was even more effective providing 95% protection [10]. Bednet coverage by young children was low at each of these sites and use of ITNs was very uncommon.

Insecticide treated materials are now the favored approach to the control of malaria in most parts of Africa and major efforts are being made to scale up their use by linking distribution to routine immunization and mass vaccination campaigns and through social marketing [11]. Provided that international support for malaria control is sustained, many countries are likely to achieve their Abuja target of 60% ITN coverage in children within the next five years. Thus, an issue that needs to be addressed urgently is whether IPT will provide significant added benefit to the imperfect protection against malaria, about 50% against clinical attacks, provided by ITNs. Previous experience in The Gambia and in Sierra Leone with the combined use of seasonal chemoprophylaxis with Maloprim ^R^  (pyrimethamine + dapsone) and ITNs suggests that it will [12,13] but this needs to be established formally.

- Project Design and Implementation

The trial will be conducted at sites in Burkina Faso and Mali. At each site, the consent of the community for their children to participate in the trial will be obtained. Once this has been given, a census of children under the age of five years will be undertaken and 3,000 will be selected at each site for inclusion in the trial. The parents or guardian of each potentially eligible child will be asked for their consent for their child to be enrolled in the study. Once consent has been obtained, each child will be examined to ensure that s/he meets the trial’s entry criteria. Children who meet the entry criteria will be given a long-lasting ITN and then be randomized to receive treatment with SP + AQ or placebo given on three occasions during the malaria transmission season.

Project staff will be placed at hospitals and health centers to which children in the trial are likely to be taken if they develop a febrile illness. A finger-prick blood sample will be taken from any child in the trial who presents at one of these designated centers with a temperature of 37.5 ^0^C or higher or a history of fever in the previous 24 hours for a rapid malaria antigen test, preparation of a blood film and collection of a filter paper sample for molecular studies. Children who are febrile and who have a positive antigen test will be treated with the nationally recommended first line treatment for malaria. Records will be kept of any study child who is admitted with severe malaria. In addition, 150 children will be visited at home each week and a blood film obtained regardless of whether or not the child is febrile to determine the impact of IPT on the prevalence of parasitemia throughout the malaria transmission season. A baseline cross-sectional survey will be conducted before treatment allocation in a random sample of children for the estimation of the prevalence of genetic markers of resistance to SP and AQ. These children will not take part in the trial. A cross-sectional survey will be undertaken at the end of the transmission season to determine the parasite prevalence rate, hemoglobin concentration of all children in the study and the prevalence of markers of resistance in children with positive parasitemia.

During the following dry season, when little malaria transmission is likely to occur, passive surveillance will be maintained at hospitals and health centers but active surveillance will be reduced to 300 visits each month. The following malaria transmission season, intensified surveillance will be re-established to determine if the combined use of ITNs and IPT had any impact on the development of naturally acquired immunity to malaria, reflected in an increased number of cases of malaria (rebound malaria) in children in the ITN + IPT group.

Clinical and laboratory methods will be standardized across sites as far as possible. An overall analysis plan will be prepared before the randomization code is broken. The trial is designed to have 90% power to detect a 20% reduction in the incidence of clinical attacks of malaria at each site in children who receive IPT in addition to an ITN. Using pooled data from each site, the trial will have 90% power to detect a 50% reduction in hospital admissions with severe malaria.

- Monitoring, Evaluation and Dissemination

The proposal will be submitted to an ethics committee at each of the trial sites and will also be reviewed by the ethics committee of the LSHTM. A Data Safety and Monitoring Board (DSMB) will be established before the trial starts to safeguard the interests of the study children. The board will receive reports on adverse events at intervals decided by the board and will review and approve the project’s analytical plan before the trial code is broken.

Once the results of the trial have been obtained, and their significance determined by the study team, the findings of the trial will be reported to the national malaria control programs of each of the countries where the studies have been done and also to other potentially interested parties such as WHO/AFRO. Results will be communicated to the population of each of the trial sites.

Results will also be presented at academic meetings and in peer-reviewed journals.

- Optimizing Public Health Outcomes

The trial proposed will be conducted in three countries where IPT might be implemented in older children as policy, if the outcome of the trial is encouraging. The fact that the research will be conducted by respected national institutions will help to ensure that the results of the trial are considered by national policy makers.

- Organizational Capacity/Management Plan

Work at each of the three study sites will be conducted under the control of the local investigators. Staff of the LSHTM will help to coordinate activities between sites and will provide logistic, technical and statistical support. A small management committee, containing representatives from each site, will help to run the project.

# **III. Goals and Objectives**

1. **Goals and Attributable Benefit**

The goal of this project is to determine the degree to which malaria can be prevented in children who sleep under an insecticide treated bednet (ITN) by the administration of intermittent preventive treatment (IPT) with an anti-malarial drug during the malaria transmission season in countries where the transmission of malaria is seasonal.

It is possible that the combination of IPT with ITNs could almost eliminate malaria as a health problem in children in areas where malaria transmission is limited to a few months of the year but, nevertheless, causes much morbidity and many deaths. At least 400 million people, including about 80 million children under the age of five years, live in the Sahel and sub-Sahel regions of Africa where malaria transmission is highly seasonal. Each year, around 400,000 of these children die from malaria and they experience at least 80 million clinical attacks of malaria a year. Thus, if the combination of IPT and ITNs provides 75% protection against malaria, as compared to the 50% provided by ITNs when used alone, and both interventions were widely implemented, the introduction of IPT could save up to 100 000 deaths and 20 million clinical attacks of malaria a year. The potential gains from this approach to malaria control are large and greater than those of IPT in infants.

**B. Objectives**

The objective of this project is to conduct an individually randomized, placebo controlled trial of IPT in children who are using an ITN to determine if IPT adds significantly to the protection against malaria provided by the ITN without adversely affecting the development of naturally acquired immunity. No previous trial has addressed these issues. The outcome of the project will be production of well validated data which establish unequivocally the impact of IPT in children who use an ITN.

IV. Project Design and Implementation

1. **Project Design**

*Introduction*

An individually randomized, placebo controlled trial of IPT with SP + AQ in children who sleep under a long-lasting ITN will be undertaken in two sites**.** The trial will be powered to give a site specific estimate of the efficacy of the combination compared with ITNs used alone in preventing clinical episodes of malaria. Results will be combined across sites for an estimate of the effect of the combination on the incidence of severe malaria.

A multi center trial is required for the following reasons –

- There are important differences in climatic and social factors between countries of the Sahel and sub-Sahel which could influence the efficacy of IPT.

- A relatively large number of subjects are required to show an effect against severe

malaria. It will be possible to recruit the number of subjects needed to meet this trial

endpoint more rapidly at multiple sites than at a single site.

- Use of several sites provides some insurance against unexpected hazards such as a

severe drought, which could be focal, or a civil disturbance which could cause the

trial to fail.

- Use of a single site might, by chance, give an unrepresentative result that had an

inappropriate influence on policy.

*Study sites and populations*

Studies will be conducted at two, strategically chosen sites situated across the sub-Sahel, the geographical area where IPT in children is likely to be most effective. These are –

*Boussé district, Burkina Faso.* Boussé is a rural area situated 40 kilometers northwest of the capital Ouagadougou. The entomological inoculation rate (EIR) is less (around 200 infectious bites per year). Most transmission occurs between July and October. At least 90% of children aged 6 – 59 months carry malaria parasites during this period. It is estimated that each child under five years of age experiences about 0.8 clinical attacks of malaria per year. ITN coverage is currently low.

*In Mali, the trial will take place in the following health centres: Djoliba, Siby and Ouelessebougou in Kati administrative region. Djoliba is located in a rural setting 60km southwest of Bamako with a population of 30000 inhabitants. Siby is located 45 km west of Bamako and the health centre covers a population of 15,232 inhabitants. Ouelessebougou is situated 80 km south of Bamako and provides service to 34,600 inhabitants*. The rainy season extends from June to October with an annual rainfall of 800 – 1200 mm. Malaria transmission is seasonal. The EIR is in the range of 100-150 infectious bites per person per season. Children have about 2 clinical attacks of malaria per year and the prevalence of parasitaemia at the end of the transmission season is about 70%. ITN usage is low.

Characteristics of the two study sites are summarized in Table 1.

**Table 1**. Characteristics of the study sites.

Variable Site

Bousse Doliba, Siby and Ouelessebougou **

Demographic 74,000 -

surveillance

Transmission June- Nov July-Nov

season

Rainfall 600 800-1200

mm/year

Net coverage in

children < 5 years

Untreated < 3% <3%

Treated < 1% <7%

Clinical attacks per 0.8 2

year in children < 5 years

Parasite prevalence 85-95% 40 - 75%

in children < 5 years

First-line treatment AL AS/AQ or AL

of malaria***

** Because of an impending malaria vaccine trial it may be necessary to move the IPTc

study to an adjacent community.

*** AQ = amodiaquine; AS = artesunate; AL = artmether/lumefantrine (Co-Artem)

*Recruitment and randomization*

After village meetings have been held in each study area to explain the nature of the trial, a census of children under five years of age resident in the study area will be conducted. At each site, approximately 3,100 children aged 3 – 59 months will be selected randomly from the census list for initial screening. The families of these children will be visited by a member of the field team and invited to participate in the study. If a family agrees that a child can join the trial, signed, informed consent will be obtained from a parent or guardian in the presence of an independent witness. Children will then be screened for their eligibility to join the trial until 3,000 have been entered. Screening will include administration of a questionnaire which asks about previous illnesses, clinical examination and measurement of height and weight.

Inclusion criteria will be –

- Age 3 – 59 months.
- Permanent residence in the study area with no intention of leaving during the surveillance period.
- Provision of informed consent.

Exclusion criteria will be -

- Temporary residence in the study area.
- Absence of informed consent.
- Presence of a severe, chronic illness such as severe malnutrition or AIDS, likely to interfere with evaluation of the trial results.
- A history of a previous, significant adverse reaction to either of the study drugs.

An acute illness, such as malaria, will not be an exclusion criterion. Such illnesses will be treated appropriately, the child randomized and retained in the trial. Children who are invited to join the trial but who are excluded for one of the reasons indicated above will be given an ITN but will not be followed further.

Children will be individually randomized using a standard computer program which gives randomization within blocks of varying number. Separate randomization lists will be generated for each site. In order to reduce the chances of the investigators becoming aware of the drug code during the course of the trial, for example because of an increased incidence of vomiting in one group, ten randomization groups will be used, five for active drugs and five for placebo. Children enrolled in the trial will be issued with a photo identity card and the parents or guardian will be asked to present this whenever their child is taken to a health centre or hospital to ensure that the child is seen by a member of the study team.

*Sample size*

The incidence of clinical malaria in unprotected children aged 3 – 59 months living in the study area detected by passive surveillance is likely to be in the range of 1.0 -2.0 clinical attacks per year and the incidence in those sleeping under an ITN will be about one half of this (0.5 to 1.0 clinical attacks per year). Assuming that children experience an average of 0.5 attacks per year of sufficient severity to present to a health facility, and the need to detect a 20% reduction in this incidence (ie. from 0.5 to 0.4 attacks per year) in children who receive IPT, the smallest reduction that would be likely to make IPT a worthwhile investment, and allowing for a 20% loss to follow-up, approximately 2,000 children (1,000 in each arm) will be required at each site to give a study with 90% power and 95% precision. A study of this size will have greater than 90% power to detect a 20% reduction in the estimated prevalence of parasitemia from 50% to 40% at the end of the rainy season. The study will have a slightly lower power to detect a 20% increase in the incidence of malaria or the prevalence of parasitemia during the second period of observation due to some loss to follow-up (likely to be in the range of 10 – 20 %). Pooling of data across sites will increase the power of the study to detect a small rebound effect.

It is estimated that the rate of hospital admissions with malaria will be in the range of 20 to 60 per 1,000. Assuming a median incidence rate of 40 per 1,000, approximately 120 admissions with severe malaria might be anticipated in children in the control arm of the study. The study will, therefore, have at least 90% power to detect a 50% reduction in admission with severe malaria (from 40 to 20 per 1,000). Initially, estimation of the impact of IPTc on admission with severe malaria was to be assessed using pooled data from three sites (MRTC, CNRFP and Navrongo Health Research Centre) and that the trial in Navrongo was suspended in June 2008 (due problems in getting ethical clearance), the number of children (2,000) who were to be enrolled at the Navrongo site has now been split between CNRFP and MRTC. Therefore the number of children to be enrolled in each of the two sites has been increased to 3,000 (1500 per arm). The study will not be powered to detect an effect on mortality but deaths will be recorded and investigated for safety reasons.

*Insecticide treated nets*

Immediately after recruitment to the trial, the mother or guardian of each study child will be provided with a long lasting, insecticide treated net (LLIN) that is marked with an identification number. Instructions will be given to the parent or guardian on how to use the net and the importance of using the net on the bed/sleeping place of the study child will be emphasized. If a child already has a net, this will be replaced and the old net returned to the family for their use. Utilisation of LLINs by study children will be monitored during home visits made to collect blood films (see below) and during the cross-sectional survey conducted at the end of the malaria transmission season.

*Intermittent preventive treatment*

Study children will be treated with a course of SP + AQ or matching placebos on three occasions during the malaria transmission season. Drugs will be pre-packaged to facilitate administration. Monthly doses will be timed to cover the peak transmission season at each site, July/August/September in Burkina Faso and Aug/Sept/Oct in Mali. Doses of AQ and SP will be based on weight as shown in Table 2. AQ will administered on the base of 10 mg/kg daily for 3 days and SP will administered as a single dose of 25mg/kg of sulphadoxine + 1.25 mg of pyrimethamine. Full tablets containing the exact daily dose (AQ) or the dose for a full course (SP) will be manufactured to avoid having to split tablets which may lead to administration of inadequate doses.

**Table 2**. Dosage of study drugs.

Weight groups Amodiaquine* SP**

Tablets (Daily dose) tablets (single dose) **

Sulfadoxine Pyrimethamine

5-9 kg 70 mg 175 mg 8.75 mg

10-18 kg 140 mg 350 mg 17.5 mg

19 kg+ 220 mg 550 mg 26.25 mg

Note. * Tablet given for 3 days;  ^**^ tablet given for 1 day

Drugs will be given under observation at a central point in the village. Children will be asked to stay at the distribution point for 30 minutes after drug administration. If vomiting occurs during this period, drugs will be re-administered. If vomiting recurs this will be noted but the drugs will not be given on a third occasion. Such children will not be excluded from the trial and they will be eligible to receive drugs at the subsequent monthly treatment. If a child misses the day set for treatment, a home visit will be made to enquire why the child was not brought for treatment and this will be recorded. If the family would like the child to continue with treatment but he or she was unable to attend on the specified day, then the first dose of treatment will be reoffered within 3 days and treatment must be completed within a maximum of 7 days after the designated date for each round. A dose missed on second of third day of treatment must be administered with 48 hours of the planned date. Thus, no child will receive two doses of SP + AQ at an interval of less than three weeks.

*Safety of IPT with SP and AQ*

Safety of SP and AQ will be monitored in all children on days 0, 1 and 2 of treatment administration and on day 3 (day after the last dose of treatment for each treatment round). .At the second and third treatment rounds, the families of each study child will be asked whether the child has had any symptoms that might have been drug related since the previous drug administration round.

Serious adverse events, including deaths and hospital admissions, will be recorded throughout the 18 months of surveillance. The likely relationship of the adverse event to drug administration will be determined according to standard criteria. Serious adverse effects will be reported to the Data Safety and Monitoring Board (DSMB) within a period specified by the board.

*Surveillance for malaria*

Surveillance for clinical attacks of malaria will start at the time of the first administration of study drugs (July/August 2007) and continue until the end of the second malaria transmission season (November 2008). Clinical episodes of malaria will be detected using a system of augmented passive surveillance. In each study area, project staff will be posted to the health centers in the area (3-5) where children in the trial are likely to go for treatment if they develop malaria. All children who attend one of these centers will be screened to determine whether or not they have been enrolled in the trial. Children identified through their identity card and/or health card as being study subjects will have their temperature recorded. A finger prick blood sample will be obtained from all study children with fever (an axillary temperature of 37.5 ^0^ C or higher) or a history of fever within the previous 24 hrs for preparation of a blood film, measurement of hemoglobin (Hb) concentration and for a rapid diagnostic test (RDT) for malaria. Those who have a positive RDT for malaria will be treated immediately for malaria according to national guidelines (AS + AQ or AL). Children with anemia will be treated according to the national protocol.

Project staff will identify any study child admitted to hospital with a possible diagnosis of malaria and ensure that their investigation includes examination of a blood film and determination of Hb concentration. The clinical features of these children will be documented on a standardized questionnaire and their clinical course followed.

To obtain more information on the impact of IPT on the prevalence of malaria infection over time in the study population, at each site a random sample of 150 children will be visited at home once a week during the peak period of malaria transmission during the first and second years of the trial. During the intervening dry season, 300 children will be visited once every four weeks. The temperature of each child will be taken and a blood film obtained regardless of whether or not a child has fever. A rapid diagnostic test will be done on any child who has fever (axillary temperature of 37.5 ^0^ C or higher) or a history of fever within the previous 24 hrs and if this is positive treatment will be given according to national guidelines (AS+AQ or AL).

At the end of the malaria transmission season, a cross sectional survey will be undertaken at which each child will be examined, their height, arm circumference and weight recorded and a finger prick blood sample obtained for determination of Hb concentration, preparation of blood films and collection of a filter paper sample for subsequent studies on the prevalence of molecular markers of resistance.

Any death in a study child will be investigated within a month of death using the post-mortem questionnaire technique.

*Surveillance for ‘rebound’ malaria*

Passive surveillance for malaria will be continued during the dry season and the subsequent malaria transmission season to determine if IPT impairs the development of natural immunity to malaria, reflected in an increase in the incidence of malaria in children who have received IPT during the previous year (rebound malaria). Identical surveillance techniques to those described above, including a final cross-sectional survey, will be used during the second year of observation.

*Surveillance for drug resistance*

Molecular markers associated with resistance to amodiaquine and to SP will be investigated in children with malaria parasitemia before and after the peak malaria transmission season to determine whether resistant parasites are selected by IPT. At baseline (before treatment administration) children will be sampled from a census database list for the prevalence of markers of resistance to SP and AQ. These children will have a clinical examination, a thick and thin blood film and filter paper blood spot prepared, and haemoglobin concentration will be determined. Samples will be collected as set out in Table 3.

**Table 3.** Samples for testing for molecular markers at each site.

Study group Number of samples Number likely to be Number for

collected positive for malaria molecular assays

Year 1

Random sample 750 300 150**

children < 5 years

before intervention *

Cross-sectional

survey – post intervention

IPT 1500 300 150**

Placebo 1500 750 150**

Year 2

Cross-sectional

survey

IPT 1500 750 150**

Placebo 1500 750 150**

Notes.

* These children will not be included in the trial but will come from the same area.

** Random sample from the positive blood samples available.

Thus, during the first year of the study approximately 450 samples will be tested from each site and during the second year 300 giving a total of 1,500 over the two years of the trial.

For the purpose of the analysis of the impact of IPT on drug resistance markers, results will pooled across sites. Sample size estimates are based on the results obtained during an IPT trial with artesunate and SP in Senegal [8]. In this trial, the prevalence of the *dhps* mutation associated with resistance to sulfonamides was 29% pre-intervention and increased post-intervention to 44% and 75% in control and intervention groups respectively. The study proposed, with 300 pre-intervention and 300 post intervention samples from placebo and intervention groups, will have over 80% power to detect a change in the prevalence of the *dhps* mutation associated with resistance of a magnitude similar to that observed in Senegal. The study will have similar power to detect a 20% difference in the prevalence of parasites carrying the triple mutation in the *dhfr* gene associated with resistance to pyrimethamine between groups at the end of the intervention period, the degree of difference between intervention and control groups seen in Senegal [8].

*Economic and social acceptability studies*

An assessment will be made of the costs of providing IPT in each of the study areas utilizing the tools developed by the economic working group of the IPTi consortium. An assessment of the acceptability of IPT will be made through focus group discussions involving the parents or guardians of study children, health care workers in the study area and local opinion leaders.

*Laboratory methods*

Thick blood films will be air dried, stained with Giemsa and examined for malaria parasites by two well-trained technicians. A hundred high power fields must be examined before a film is declared negative. Parasite density will be determined by counting the number of parasites present per white blood cell (WBC) and assuming a WBC count of 8,000 per ul. In the case of discrepant positive/negative results being obtained a third, experienced reader will be asked to read the film and his/her result will be considered definitive. If parasite densities differ by more than 30%, a third experienced reader will also be asked to examine the film; the two closest readings will be used to determine the mean. Prior to the start of the trial, blood films will be exchanged between study sites and read at each site to identify any inconsistencies in microscopic techniques. During the course of the study, 500 randomly selected slides obtained from each site will be read at each of the other sites each year to ensure consistency of readings across sites.

Hemoglobin concentrations will be measured in the field using a Hemocue (Hemocue AB, Angelholm, Sweden). Assays of molecular markers associated with resistance to amodiaquine and SP will be determined by mutation specific PCR and/or nested PCR followed by restriction digestion as previously described: DHFR Ser®Asn 108, Asn®Ile 51,  Cys®Arg 59, and Ile®Leu 164 (Plowe 1995, Djourte 1999, Doumbo 2000); DHPS Ala®Gly 437, and Lys®Glu 540 (Wang 1997); PfCRT K76T; pfmdr1 N86Y (Djimde 2001). Detailed protocols for these methods are available at [http://medschool.umaryland.edu](http://medschool.umaryland.edu/) /CVD/plowe.html. Samples from each site will be tested centrally at MRCT, Bamako.

*Definitions*

The following definitions will be used during the course of the study.

*Clinical malaria*. The primary definition of malaria will require:

(a) the presence of fever (axillary temperature > 37.5 ^o^ C) or a history of fever in the past

24 hours,

(b) the absence of any other obvious cause of the fever,

(c) the presence of *P. falciparum* asexual parasitemia above a threshold value of 5,000

parasites per ul (a threshold shown previously to be of value in differentiating

symptomatic malaria from other illnesses associated with co-incidental

parasitemia).

A secondary definition of malaria will require only the presence of *P. falciparum* parasitemia at any density.

*Severe malaria*. Severe malaria will be defined according to the WHO criteria [Trans R Soc Trop Med Hyg 2000;94 suppl 1]. Joint training of staff from each site will be undertaken to ensure standardization in the recording of symptoms and signs that are used to make a diagnosis of severe malaria before the trial commences.

*Anemia*. Anemia will be defined as an Hb concentration <11 g/dl, moderate anemia as an Hb concentration < 8 g/dL and severe anemia as an Hb concentration < 5 g/dl.

*End-points*

The primary end-point for the first and second years of the study will be the incidence of clinical malaria, detected by passive case detection, defined as above.

Secondary endpoints will be –

(a) the incidence of clinical malaria using a definition that does not require a parasite

density threshold,

(b) the incidence of severe malaria as defined above,

(c) the incidence of anemia among children seen at a hospital or health centre,

(d) the prevalence of parasitemia at the end of malaria transmission season cross-

sectional survey,

(e) the prevalence of anemia at the end of malaria transmission season cross-sectional

survey,

(f) the prevalence of stunting and the proportion of children underweight at the end of

malaria transmission season cross-sectional survey,

1. the proportion of children with parasites carrying resistance markers to amodiaquine, SP

or sulfonamides at the end of transmission season cross-sectional survey,

1. the incidence of all cause hospital admissions,

(i) the incidence of hospital admissions due to malaria,

(j) the number of malaria treatments received by each child.

*Data management and analysis*

As far as possible, identical forms, in English or French, will be used at each site. Data will be double entered at each site and validated. An individual analytical plan will be prepared for each site together with an analysis plan for the combined data from the three sites. These plans will be submitted for approval by the DSMB established for the trial before the code is broken. Breaking the code and initial analysis of the trial results will be done at a meeting of the investigators.

*Ethics*

The study protocol will be submitted for approval to the appropriate ethics committee(s) at each of the trial sites. In Burkina Faso this will be the Ethics Committee for Research, in Ghana, the Health Service Ethical Review Committee and the Navrongo Health Research Centre Institutional Review Board and in Mali, the ethical committee of the Faculty of Medicine Pharmacy and Dentistry of the University of Bamako (US, DHHS/OHRP Federal Wide Assurance #: FWA00001769). The protocol will also be reviewed by the ethics committee of the London School of Hygiene & Tropical Medicine.

*Timetable*

**2008**

April - June Preparation of trial sites.

July - Nov Administration of IPT and surveillance for malaria.

December Laboratory analyses.

**2009**

Jan – June Continuation of laboratory analyses.

Data cleaning.

July – Nov Surveillance for ‘rebound’ malaria.

December Laboratory analyses.

**2010**

Jan – March Analysis and reports.

1. **Major Activities and Milestones**

To meet the objectives of the project four major activities will be conducted.

*Activity 1*

Conduct of a randomized, placebo controlled trial to determine the impact of adding IPT to the use of ITNs on the incidence of uncomplicated malaria and severe malaria in 6,000 children at two sites in Africa.

The target output for this activity will be a well validated data set which establishes whether IPT with SP + AQ provides added benefit to ITNs in preventing uncomplicated and/or severe malaria.

*Activity 2*

Follow-up of 6,000 children at two sites in Africa enrolled in the randomised trial identified as activity 1 to determine if adding IPT to the use of ITNs impairs the development of natural immunity to malaria reflected in an increase of cases of clinical malaria in the year following treatment.

The target output for this activity will be a well validated data set which establishes whether adding IPT to the use of ITNs impairs the development of natural immunity to malaria.

*Activity 3*

Coordination of activities across study sites to ensure compatibility of data collection at each site and facilitation of analysis.

The target output for this activity will be a well coordinated, multi-center trial.

Milestones for each site, indicated in Appendix A, will include –

- Meetings in the study communities to explain the purpose of the trial (May 2008)
- Census of children in study villages, selection of eligible children for enrollment and

collection of individual consent by the start of the 2008 malaria transmission

season (July 2008).

- Conduct of a randomized trial of IPT in children using an ITN by December 2008.

This will involve net distribution, drug administration and surveillance for

uncomplicated and severe malaria.

- Data cleaning and laboratory analysis, including molecular typing of drug

resistance markers, of samples obtained during the previous rainy season at each

site by May 2009.

- Further surveillance of all available children for a second malaria transmission

season at each site to detect possible rebound malaria by December 2009.

- Analysis of results and presentation of preliminary finding to the local health

authorities and the study populations by March 2010.

1. Challenges

The main risk to this study is a severe drought which reduces malaria transmission to such an extent that there are insufficient cases of malaria to provide a clear result. This risk is mitigated to some extent by having three sites, each powered to give an individual result, as droughts in the Sahel are often focal. However, the risk is not completely prevented by utilizing three sites as a very severe drought might affect countries across the Sahel and sub-Sahel. A second risk is that a civil disturbance at a critical time of the year could interfere with field work. This is unlikely as the three countries selected are currently stable, but this possibility cannot be excluded.

Technical concerns include the possibility that IPT might be perceived to be harmful, leading to poor compliance. The SP + AQ combination causes minor side effects in some children and these could be perceived as being more serious than they are. A sound program of education about the trial, conducted in each site, before it starts should help to reduce this risk.

V. Monitoring, Evaluation, and Dissemination

To ensure that the project is conducted to the highest ethical standards, the proposal will be reviewed by ethics committees at each study site and at LSHTM. To ensure that the interests of study children are well protected, a DSMB will be established to monitor the trial. All serious adverse events and grade 3 adverse events which might be drug related will be reported to the DSMB, and the relevant ethics committee, within a time period agreed by the DSMB. Other adverse events will be summarized by group and reported to the DSMB at a frequency decided by the board. The DSMB will be supported by a local safety monitor at each site.

To ensure that the trials are conducted to the standards of Good Clinical Practice (GCP), a trials monitor will be appointed. He or she will visit each site before the trial starts, on at least one occasion during the collection of data and at the end of the trial.

To ensure that the trial meets its scientific targets a management group will be established which will have representatives from each partner. This will meet face to face before the trial starts and at least once a year subsequently. More frequent contact will be made through teleconferences. The purpose of the Management Group will be to facilitate interactions between sites, ensure that each site achieves its targets and to arrange technical or logistic support to any site, should this be needed.

Once the data from each trial have been analyzed according to a predefined analytical plan approved by the DSMB, a meeting of the partners, including representatives from the national malaria control program, will be held at which the implications of the findings will be discussed. Once an agreement on the findings and their policy implications has been reached, the results of the study will be presented to the Ministries of Health of each of the host countries and to the communities where the trials have been conducted. Results will be prepared for publication and presented in peer-reviewed international journals, probably as findings from each site together with an overview.

VI. Optimizing Public Health Outcomes and Intellectual Property Plans to Achieve Global Access

1. **Overview**

Several factors will help to ensure that the results of this study are available in the communities where they will be of most practical value. These are -

- The study will be conducted in three countries which are potential sites where IPT in children might be implemented, if results from the trial are promising.
- The studies will be done by respected national institutions whose results are likely to be accepted by national and regional health authorities.
- A dissemination plan will be drawn up before the trial is completed that ensures that the results of the trial are made available in an appropriate format for presentation to relevant national authorities and to groups such as WHO.

1. **Optimizing Public Health Outcomes**

If the results of the proposed trial indicate that the addition of IPT to the use of ITNs provides significant added protection against malaria without any serious side effects or impact on the development of immunity, this approach will require serious consideration as a malaria control tool in areas where malaria transmission is seasonal. However, additional information may be required before large scale implementation could be recommended. This includes determining whether the intervention could be delivered on a large scale and whether IPT given for several years might impair immunity or have a deleterious effect on the prevalence of drug resistance. The first of these issues is being addressed by studies in Ghana, The Gambia and Senegal but further studies may be needed in the countries involved in this trial. Determination of the latter will require several years of observation, perhaps within the context of a demonstration project.

Staff of the national malaria control programs of the countries where the trials are being done and other interested parties, such as WHO/AFRO and the Global Malaria Partnership, will be kept informed of the aims of the study and of its progress so that, if the outcome is very positive, there will be no unnecessary gap between completion of the study and discussions on the possible implementation of IPT in children.

VII. Organizational Capacity and Management Plan

1. **Organizational Capacity and Facilities**

Each member of the partnership has the experience and skills necessary to ensure that the study is conducted successfully.

*LSHTM*

The Malaria Centre at LSHTM has an established record of working successfully with partners in Africa on clinical trials. During the past five years, staff of the Malaria Centre have worked with colleagues from Africa on over 20 trials of malaria treatment or prevention including studies of intermittent preventive treatment in infants in Ghana (3) and in children in Senegal (8) and Ghana. Staff of the Malaria Centre at LSHTM have previously collaborated successfully with CNRFP in Burkina Faso

*CNRFP*

The *Centre National de Recherche et de Formation sur le Paludisme* (CNRFP) is a department of the Ministry of Health of Burkina Faso. The mandate of CNRFP is to (i) participate in the formulation, implementation, supervision, and evaluation of malaria control activities in collaboration with the national malaria control program, (ii) to carry out operational and basic research to identify new malaria control strategies and adapt existing ones to the local conditions and (iii) to provide training to health staff and scientists from Burkina Faso. Since its foundation in 1983, CNRFP has developed research collaborations with many southern and northern research institutions. The CNRFP team consists of scientists with skills in several disciplines, several post-doctoral fellows and graduate students. Malaria research and control activities conducted at CNRFP include studies of the epidemiology of malaria, community-based interventions, prevention of malaria in pregnancy, clinical trials of anti-malarial drugs or drug combination and vaccines. CNRFP is currently running two separate demographic surveillance sites with total populations of about 200,000 inhabitants.

*MRTC*

The Malaria Research and Training Center (MRTC), University of Bamako, Mali was created in 1992 within the Department of Epidemiology of Parasitic Diseases (DEAP), at the Faculty of Medicine, Pharmacy and Odonto-Stomatology (FMPOS), University of Bamako through several partnerships and grants. It is one of the strongest malaria research institutions in Africa, with support from numerous funding agencies and collaborative relationships with several northern and southern research institutions. The center in Bamako supports several field sites. Each field site has its research team comprising medical doctors, pharmacists, pre-doctoral medical students and local facilitators under the supervision of senior scientists. The research scope of the center includes clinical trials, clinical and molecular parasitology, immunology, pathogenesis, drug resistance, basic and molecular epidemiology, basic and molecular entomology and geographic information systems and remote sensing. Staff of the center have undertaken several intervention studies including trials of the efficacy of impregnated bed nets, intermittent preventive treatments in pregnant women and children and malaria treatments and vaccines trials.

**Management and Staffing Plan**

Each trial will be conducted under the direction of its own PI(s) and will be fully independent. LSHTM will help with co-ordination, technical advice and logistic support. Study design and methods will be coordinated through the work of a small management committee which will include representatives from each site. This will meet before the trial starts and at least once a year thereafter. Regular contact will be maintained through teleconferences. An initial workshop to standardize methods and instruments will be held and PIs will be encouraged to visit each other’s sites. Regular visits to trial sites will be made by staff of the coordinating group at LSHTM.

VIII. Budget Narrative

Costs of the trial include contributions to the salaries of senior investigators and support for the clinical officers, field staff and laboratory staff who will conduct the trial at each site. Support for a trial coordinator, who will be based at LSHTM is also requested. S/he will be responsible for co-ordination of standard operating procedures, data collection and data entry between sites. Capital costs include funds for a new 4 x 4 vehicle at each site (this will be supplemented by existing transport) motorcycles, microscopes and generators. The latter are required to support microscopy at the health centers where study patients attend for treatment which do not already have this facility. A long-lasting insecticide impregnated bednet will be needed for each study child. Consumable costs will be those required to subsidize patients’ care in the clinic and those for RDTs, Hb measurements and microscopy. A large number of molecular assays will be required to detect any possible impact of IPT on the prevalence of resistance markers. It has proven difficult so far to identify a manufacturer who is willing to produce SP and amodiaquine and matching placebo so the budget line for this item, which includes packing, is only an estimate based on costs obtained in previous studies. The project will require substantial field work and funds will be required to support local travel. Requests for international travel cover travel between sites and an annual meeting of all investigators.

In order to obtain as economical a price as possible, ITNs, RDTs and Hemocue cuvettes will be purchased centrally by LSHTM. Conducting all molecular assays in Mali will also help to reduce costs.

As has been agreed with Foundation staff, all partners are claiming indirect costs on modified direct costs at the lower rate of 10%.

**J. Support for Proposed Project from Other Sources**

| **Other Donor** | **Amount**  **U.S.$** | **% of project** | **Committed or Potential** |
| --- | --- | --- | --- |
| LSHTM  (staff related costs for  B. Greenwood & D. Chandramohan) | $93,944 | 3.14 | Committed |

IX. Citations

1. Lengeler C. Insecticide-treated bed nets and curtains for preventing malria. Cocchrane Database Systematic Reviews 2004; CD000363.

2. Schellenberg D et al. Intermittent treatment for malaria and anaemia control at time of routine

vaccination in Tanzanian infants; a randomised, placebo-controlled trial. Lancet 2001 ;357 :1471- 77.

3. Schellenberg D et al. Intermittent preventive antimalrial treatment for maalria in Tanzanian infants;

follow-up to age to 2 years of a randomised, placebo-controlled trial. Lancet 2005; 365:1481.

4. Massaga et al. Effect of intermittent treatment with amodiaquine on anaemia and malaria fevers in

infants in Tanzania:a randomised , placebo-controlled trial. Lancet 2003; 361:1853-60.

5. Chandramhohan D et al. Prevention of malaria in infants by intermittent preventive treatment in an

area of high seasonal transmission in Ghana. BMJ 2005; 331:727-33.

6. Macete E. et al. Intermittent preventive treatment for malaria control administered at the time of

routine vaccinations in Mozambican infants: a randomized, placebo-controlled trial. J Infect Dis

2006;194:276-85.

7. Taylor T, et al. Standardized data collection for multi-center clinical studies of severe malria in African

children:establishing the SMAC network. Trans R Soc Trop Med Hyg 2006;100:615-22.

8. Cisse B et al. Seasonal intermittent preventive treatment with artesunate and sulfadoxine

pyrimethamine prevents malaria in Senegalese children. Lancet 2006; 659-67.

9 Dicko A et al. Impact of intermittent preventive treatment with sulfadoxine-pyrimethamine targeting the

transmission season in the incidence of clinical malaria in children aged 6 months to 10 years in

Kambila, Mali. Amer J Trop Med Hyg 2004;71: suppl S4, 6.

10. Sokhna C et al. A trial of the efficacy, safety and impact on drug resistance of four drug regimens for

seasonal intermittent preventive treatment for malaria in Senegalese children. Submitted to Lancet.

11. Hill J, Lines J, Rowland M. Insecticide-treated nets. Advances in Paraistology 2006;61:78-128.

12. Alonso P et al. A malaria control trial using insecticide-treated bed nets and targeted

chemoprophylaxis in a rural area of The Gambia, West Africa. 6. The impact of the interventions on

mortality and morbidity from malaria.Trans R Soc Trop Med Hyg 1993; 87 suppl 2: 37-44.

13. Marbiah NT et al. A controlled trial of lambda-cyhalothrin-impregnated bed nets and/or

dapsone/pyrimethamine for malaria control in Sierra Leone. Amer J Trop Med Hyg 1998;58:1-6.

**X. Appendices**

*Appendix materials do not count against page limits. Please do not include additional appendices beyond the materials requested under sections A-D below unless requested by Global Health program staff.*

**A. Milestone Summary Table and Timeline**

See attached**.**

B. Budget Spreadsheet

See attached.

**C.** Financial and Tax Information

1. If your organization is a U.S. public charity, please include a copy of the organization’s most recent 990-tax form. For all other organizations, please include the most recent financial statement. If this is not available electronically, please fax separately to 206-709-3170, using a copy of the full proposal summary information page as the coversheet.

2. If your organization is a public charity, private foundation, government unit, non-charitable tax-exempt organization, or international organization by executive order, please include an electronic copy of the valid IRS determination letter. If this is not available electronically, please fax separately to 206-709-3170, using a copy of the proposal summary information page as the coversheet.

LSHTM Financial Statement 2004-2005 is attached as an annex.

1. **Biographical Information**

**Name: DIALLO Diadier**

**Project role:** Project leader in Burkina Faso.

**Positions Held:**

Current position**:** Researcher at CNRFP.

1998-2001: Responsible for the epidemiology and data management unit at CNRFP.

*During this period I was responsible for the conduct and analysis of a number of*

*studies on the epidemiology of malaria including a long-term follow-up of children*

*who had slept in rooms protected by insecticide-treated curtains*.

1994-1997 Responsible for the data management unit at CNRFP.

**Education:**

2001-2005: PhD at the London School of Hygiene and Tropical Medicine, United Kingdom.

1997-1998: Master of Science in Communicable Disease Epidemiology at the London School of Hygiene and Tropical Medicine (United Kingdom).

1994-1995: Master of Science Biochemistry and Microbiology, University of Ouagadougou (Burkina Faso).

1986-1990: Diploma in Medical Laboratory Sciences, National University of Benin, Cotonou (Benin).

**Representative publications:**

**Diallo D. A**., Cousens S. N., Cuzin-Ouattara N., Nebie I., Ilboudo-Sanogo E. & Esposito F. (2004). Child mortality in a West African population protected with insecticide-treated curtains for a period of up to 6 years. *Bulletin of the World Health Organization* **82**: 85-91.

Meraldi V., Nebié I., Tiono A.B., **Diallo D. A**., Sanogo E., Theisen M., Druile P., Corradin G., Moret R.,.& Sirima B.S. (2004). Natural antibody response to *Plasmodium falciparum* Exp-1, MSP-3 and GLURP long synthetic peptides and association with protection. *Parasite Immunology* **26**: 265-72.

Nebié I., Cuzin-Ouattara N**., Diallo D. A**., Cousens S. N., Theisen M., Corradin G., Traoré A.S.& Esposito F. (2003). Humoral responses to defined malaria antigens in children living since birth under insecticide-treated curtains in Burkina Faso. *Acta Tropica* **88**: 17-25

Sanogo-Ilboudo E., Cuzin-Ouattara N., **Diallo D.A.**, Cousens S.N., Esposito F., Habuetzel A., Sanon S. & Ouédraogo A.P. (2001). Insecticide treated materials, mosquito adaptation and mass effect: entomological observations after five years of vector control in Burkina Faso. *Transactions of the Royal Society of Tropical Medicine and Hygiene,* (in press).

Muller O, Becher H, van Zweeden AB, Ye Y, **Diallo DA**, Konate AT, Gbangou A, Kouyate B, Garenne M. (2001) Effect of zinc supplementation on malaria and other causes of morbidity in west African children: randomised double blind placebo controlled trial. *British Medical Journal* ;322:1567.

**Name: DICKO Alassane**

**Project Role:** Project director in Mali

**Positions held :**

Jan 03 –Present Associate Professor and Head of Epidemiology, Biostatistics & Data

Management Unit, Malaria Research & Training Center, Department of

Epidemiology of Parasitics Diseases, Faculty of Medicine, Pharmacy and

Dentistry, University of Mali.

*I am the Doneguebougou trial site coordinator and investigator in various studies including trials of malaria vaccines and drug testing. I also supervise the data management team staffed with 5 technicians and three MD, MPH and one MPH. My other duties include teaching epidemiology to medical students as well as the training and mentoring of students and research assistants.*

Jun 01-Jan 03 Research Associate and Head of Epidemiology, Biostatistics & Data Management Unit, Malaria Research & Training Center, Department of Epidemiology of Parasitics Diseases, Faculty of Medicine, Pharmacy and Dentistry, University of Mali.

*I was coordinator and investigator on studies undertaken in preparation for malaria vaccine testing in Bandiagara. I was in charge of the data management, analysis and quality control. Other duties include training and mentoring of students and research assistants.*

Apr 98 – Apr 01 Visiting/Research Fellow, Center for Vaccine Development, University of Maryland, USA & Laboratory of Parasitic Diseases, NIAID/NIH, Bethesda, MD, USA

*During this fellowship, I received training in vaccinology, molecular epidemiology, design of studies, writing protocols, supervising data collection, Good Clinical Practice and Laboratory Practices.*

Oct 94- Apr 98 Researcher, Malaria Research & Training Center, Department of Epidemiology of Parasitic Diseases, Faculty of Medicine, Pharmacy and Dentistry, University of Mali

*I was the field team leader in charge of the conduct of several epidemiological studies at different studies sites,*

Oct 93- Oct 94 Research Assistant, Malaria Research & Training Center, Department of Epidemiology of Parasitic Diseases, Faculty of Medicine, Pharmacy and Dentistry, University of Mali

*I was a member of a field team conducting epidemiological studies on malaria (epidemiological indicators and drug resistance).*

### Education

1994 MD National School of Medicine and Pharmacy, Bamako, Mali

1997 Certificates in Biostatistics, John Hopkins University, Baltimore, USA

1. MSc in epidemiology, University of Maryland, Baltimore, USA
2. Certificate for clinical monitors. Association of clinical research professionals

2005 Certificate of good clinical practice for trial monitors. WHO, TDR

**Representative publications**

1. Sissoko MS, **Dicko A**, Briet OJ, Sissoko M, Sagara I, Keita HD, Sogoba M, Rogier C, Toure YT, Doumbo OK. Malaria incidence in relation to rice cultivation in the irrigated Sahel of Mali. *Acta Trop. 2004;89:161-70.*
2. Lyke KE, **Dicko A**, Kone A, Coulibaly D, Guindo A, Cissoko Y, Traore K, Plowe CV, Doumbo OK. Incidence of severe *Plasmodium falciparum* malaria as a primary endpoint for vaccine efficacy trials in Bandiagara, Mali. *Vaccine. 2004;22:3169-74.*
3. **Dicko A**, Klion AD, Thera MA, Sagara I, Yalcouye D, Niambele MB, Sogoba M,Dolo G, Dao A, Diallo DA, Doumbo OK, Miller LH. The etiology of severe anemia in a village and a periurban area in Mali. *Blood. 2004;104:1198-200. Epub 2004 Apr 27*.
4. **Dicko A,** Mantel C, Kouriba B, et al. Season, fever prevalence and pyrogenic threshold for malaria disease definition in an endemic area of Mali. *Trop Med Int Health. 2005; 10:550-6.*
5. Sagara I, **Dicko A**, Guindo O, Djimde A, Thera MA, Sogoba M, Kone M, Fofana M, Ouattara A, Baby M, Sissoko M, Jansen HF, Doumbo OK. An opened randomized clinical trial comparing comparing CO-ARINATE® (Artesunate plus Sulfamethoxypyrazine-pyrimethamine) versus COARTEM® (Artemether plus Lumefantrine) in the treatment of uncomplicated Plasmodium falciparum malaria in Mali.  *Am J Trop Med Hyg, in press.*

**Name: GREENWOOD Brian M**

**Project role: Coordinator**

**Positions held:**

1. Manson Professor of Clinical Tropical Medicine, London School of Hygiene and

Tropical Medicine, London, UK.

At LSHTM, I set up the Malaria Centre and directed this until 2005.

From 2000, I have been director of the multi-center Gates Malaria Partnership.

The latter has involved supervising a broad based research program, providing

support for post-doctoral and PhD students and providing support to the training

centers in Africa established under the program.

1996-8 Professor of Communicable Diseases, London School of Hygiene and Tropical

Medicine, London, UK.

1980-95 Director of the Medical Research Council Laboratories, The Gambia.

*In the Gambia, I directed a large multi-disciplinary research program directed at*

*the control of infectious diseases of major importance in West Africa.*

**Education:**

1959 B.A. Natural Sciences, University of Cambridge, UK.

1962 M.A. Natural Sciences, University of Cambridge, UK.

M.B., B. Chir., University of Cambridge, UK.

1969 M.D., University of Cambridge, UK.

**Representative publications:**

Chandramohan D, Owusu-Agyei S, Carneiro I, Awine T, Ampona-Achiano K, Mensah N, Jaffar S, Baiden R, Hodgson A, Binka F, **Greenwood B**. Cluster randomised trial of intermittent preventive treatment for malaria in infants in area of high, seasonal transmission in Ghana. British Medical Journal 2006; 331: 727-33.

Cisse B, Sokhna C, Boulanger D, Milet J, Ba EH, Richardson K, Hallett R, Sutherland C, Simondon K, Simondon F, Alexander N, Gaye O, Targett G, Lines J, **Greenwood B M**, Trape J-F. Seasonal intermittent preventive treatment with artesunste and sulfadoxine-pyrimethamine for prevention of malaria in Senegalese children: a randomised, placebo-controlled, double-blind trial. Lancet 2006;367:659-67.

**Greenwood BM**. The use of anti-malaria drugs to prevent malaria in the population of malaria-endemic areas. American Journal of Tropical Medicine and Hygiene 2004; 70: 1-7.

**Greenwood B**. Intermittent preventive treatment – a new approach to the prevention of malaria in children in areas with seasonal malaria transmission. Tropical Medicine and International Health 2006, 11: 983-91.

**Greenwood BM**, Bojang K, Whitty CJM, Targett GATT. Malaria. Lancet 2005; 365: 1487-98.

XI. Additional Required Narrative for Compliance with Regulatory Guidelines

*Sections A.1-3 apply only to projects involving research. Section A.4 may apply to research and/or demonstration projects.*

1. Research Assurances

The project will not involve any research on animals.

The project will involve a major clinical trial.

*Ethics:* The proposal will be reviewed by ethics committees In Burkina Faso, Ghana and Mali as specified above. The protocol will also be reviewed by the ethics committee of the London School of Hygiene & Tropical Medicine.

*Insurance:* Insurance of trial participants and of the staff conducting the trial will be covered by a comprehensive insurance scheme held by LSHTM for all clinical trials for which it is the sponsor.

*Registration:*  The trial will be entered on the Clinical trials Register held by the National Institutes of Health.

*Regulatory authorities:* Only licensed drugs will be used so approval from the national regulatory authorities will not be required.

**London School of Hygiene and Tropical Medicine**

**Study title**

**A trial of the combined impact of intermittent preventive treatment and insecticide treated bednets on morbidity from malaria in African children**

**Trial Director**

Prof Brian Greenwood, LSHTM ([Brian.Greenwood@lshtm.ac.uk](mailto:Brian.Greenwood@lshtm.ac.uk))

**Trial Coordinator**

Diadier Diallo, LSHTM ([Diadier.Diallo@lshtm.ac.uk](mailto:Diadier.Diallo@lshtm.ac.uk)

**Site Principal investigators**

Alassane Dicko, MRTC Mali ([adicko@MRTCBKO.org](mailto:adicko@MRTCBKO.org))

Amadou T Konate, CNRFP, Burkina Faso ([a.konate.cnlp@fasonet.bf](mailto:a.konate.cnlp@fasonet.bf))

_________________________________________________________________________

**Protocol amendments**

**Date 20^th^ June 2008**

**Amendments to version 1 of the protocol (20^th^ June 2008)**

**Summary of changes made**

The trial steering committee comprising staff from the London school of Hygiene and Tropical medicine and the trial Principal investigators at the sites in Africa met in March and June 2008 and made the following amendments to the protocol the version of the protocol as described below. These amendments will be submitted to Ethics committee of institutions participating in the trial.

1. Dosage of sulfadoxine-pyrimethamine and amodiaquine

It was indicated in the protocol that sulphadoxine-pyrimethamine (SP) and amodiaquine (AQ) dosage will be based on child’s age. Four age categories were constituted. However, considering the difficulty of correctly estimating child’s age in rural communities, the study steering committee agreed that drug administration based on child’s weight will be more appropriate in routine programme settings. Therefore three weight groups have been constituted (5-9 kg; 10-18 kg and 19 kg and over). The exact daily dose (AQ: 70mg for 5-9kg, 140 mg for 10=18kg and 220 mg for 19+kg; SP: 175 mg + 8.75 mg for 5-9 kg; 350 mg +17.5 mg for 10-18 kg and 550 mg + 26.75 mg for 19+kg) will be manufacturing for each weight to avoid breaking tablets in halves or quarters, which may results in inadequate dosage of drugs.

1. Trial sites

Ethical clearance was obtained for the conduct of the study in Burkina Faso and Mali. The third trial site (Navrongo Health Research Centre) was not successful in getting ethical clearance from the Ghana Food and Drug Board (FDB). The FDB raised some queries which the site is currently addressing. However, it is feared that obtaining ethical clearance after re-submission of a revised protocol to the FDB is likely to take at least 2 months which will cause a significant delay in the implementation of the study. Therefore it was agreed to suspend the trial in Navrongo. Consequently, sections relating to the Navrongo Health Research Centre have been removed from the protocol.

1. Randomization

The first version of the protocol indicates that randomisation will be done in 8 blocks (4 blocks per group). To minimise risk of “unblinding” of research staff, the number of blocks was increased to 5. Thus, randomization will now be done in permuted blocks of 10 (5 blocks per group).

1. Samples size

4.1 The number of children to be enrolled in the trial in Navrongo has been split between MRTC Mali and CNRFP, Burkina Faso as the trial will now be conducted in these two sites only. The number of children to enrolled at each of these two sites is now increased to 3,000 children (1,500 per arm). The initial sample size of 2000 children per site was estimated to have adequate power to measure the protective efficacy of IPTc on malaria morbidity at each site and to pool the data from all three sites (6,000 children) to measure the impact of IPTc on admission to hospital with severe malaria. Therefore, in order to have adequate power to measure the effect of IPTc on hospital admissions with malaria, we have to increase the sample size from 2,000 to 3,000 in Burkina Faso and Mali.

4.2 The number of children to be enrolled in the weekly and the monthly surveys for the effect of IPTc on the prevalence of malaria infection and the number of samples to be analysed per site to assess the impact of IPTc on the prevalence of markers of resistance to SP and AQ have been re-adjusted by redistributing samples that were to be collected at the Navrongo site between CNRFP and MRTC. Thus site will collect 2500 more samples over the study period (see table 3 below).

1. Baseline survey of markers of resistance to SP and AQ

A random sample of children from the study villages’ census lists will be selected for the baseline survey of the prevalence of markers of resistance to SP and AQ (about 400-500 children per site). Children who will be involved in this survey will not take part in the trial of intermittent preventive treatment, but will be given and ITN. Initially, these children were to be recruited from neighbouring villages.

1. Safety of SP and AQ (Monitoring of adverse events)

The initial plan was to conduct weekly visits in a random sample of 100 children to monitor adverse events. It has now been amended that adverse events will be assessed on days 0, 1 and 2 of IPTc administration and the day after the last dose of IPTc administration in all children enrolled in the study. Adverse events that may occur after day 3 post IPTc will be recorded on day 0 of the next round of IPTc administration. This option provides the opportunity for monitoring adverse events in all study children.

1. Intermittent Preventive Treatment administration

The revised version of the protocol specifies that children who miss the first dose of IPTc administration of a given treatment round must be visited within 3 days of the planned date of visits instead of 7 days as mentioned in the previous version of the protocol. The three doses of each IPTc course must be completed within 7 days of starting the first dose of IPTc In addition, children who miss the second or the third dose of each IPTc course must be seen within 48 hours. The reason for this approach is to make sure that the interval between IPTc courses is at least 21 days.

1. Surveillance of malaria morbidity

The previous version of protocol indicates that filter paper samples will be taken from sick children to assess markers of resistance to SP and AQ. The steering committee has decided not to take filter paper samples from sick children because the value of these samples to understand the effect of IPTc on drug resistance markers is questionable. Furthermore the study doses not have adequate resources to collect these samples and to analyse them.

1. End of malaria transmission season cross-sectional survey

The previous version of the protocol indicates that weight and height were the anthropometric parameters to be measured during the survey. However after discussion with an anthropologist it was suggested to measure child’s arm circumference during this survey. Now the protocol is amended that mid arm circumference will also be measured during child’s screening for enrolment in the trial to provide baseline information.

1. Slide reading

The previous version of the protocol stated that 100 high power field were to be examined before a slide is declared negative. According to the revised protocol all fields in a slide must be examined before a blood smear is declared negative for malaria parasites

**Implementation of protocol amendments**

Amended sections are presented in italics and the precise location of the amendment is underscored. The section on top is the section as it appears in the non amended version of the protocol.

.

**II. Executive summary**

**Project design and implementation (page 4)**

“The trial will be conducted at sites in Burkina Faso, Ghana and Mali. At each site, the consent of the community for their children to participate in the trial will be obtained. Once this has been given, a census of children under the age of five years will be undertaken and 2,000 will be selected at each site for inclusion in the trial.

*Amendment*

*“The trial will be conducted at sites in Burkina Faso and Mali. At each site, the consent of the community for their children to participate in the trial will be obtained. Once this has been given, a census of children under the age of five years will be undertaken and 3,000 will be selected at each site for inclusion in the trial”.*

**Project design and implementation, page 4 last paragraph (lines 7-13)**

“…Records will be kept of any study child who is admitted with severe malaria. In addition, 100 children will be visited at home each week and a blood film obtained regardless of whether or not the child is febrile to determine the impact of IPT on the prevalence of parasitemia throughout the malaria transmission season. A cross-sectional survey will be undertaken at the end of the transmission season to determine the parasite prevalence rate and hemoglobin concentration of all children in the study”.

*Amendment*

*“….Records will be kept of any study child who is admitted with severe malaria. In addition, 150 children will be visited at home each week and a blood film obtained regardless of whether or not the child is febrile to determine the impact of IPT on the prevalence of parasitemia throughout the malaria transmission season. A baseline cross-sectional survey will be conducted before treatment allocation in a random sample of children for the estimation of the prevalence of genetic markers of resistance to SP and AQ. These children will not take part in the trial. A cross-sectional survey will be undertaken at the end of the transmission season to determine the parasite prevalence rate, hemoglobin concentration of all children in the study and the prevalence of markers of resistance in children with positive parasitemia”*.

**Project design and implementation, page 5, paragraph 1, sentence 1.**

“During the following dry season, when little malaria transmission is likely to occur, passive surveillance will be maintained at hospitals and health centers but active surveillance will be reduced to 100 visits each month”.

*Amendment*

*“During the following dry season, when little malaria transmission is likely to occur, passive surveillance will be maintained at hospitals and health centers but active surveillance will be reduced to 300 visits each month”.*

**IV. Project design and implementation (page 6)**

1. **Project design**

Introduction

A multi-center, individually randomized, placebo controlled trial of IPT with SP + AQ in children who sleep under a long-lasting ITN will be undertaken**.**

*Amendment*

*“An individually randomized, placebo controlled trial of IPT with SP + AQ in children who sleep under a long-lasting ITN will be undertaken in two sites”***.**

***Study sites and populations***

“Studies will be conducted at three, strategically chosen sites situated across the sub-Sahel, the geographical area where IPT in children is likely to be most effective. These are –

*West Mamprusi district, Ghana.* West Mamprusi district is situated in an area of open savanna in northern Ghana with a population of around 132,000. Annual rainfall is around 850 mm per year. The malaria transmission season last from July to October; during this period residents may receive as many as 400 infectious bites. Children experience about 1 clinical attack of malaria a year. ITN usage is currently modest – about 30% in children under the age of five years.

*Boussé district, Burkina Faso.* Boussé is a rural area situated 40 kilometers northwest of the capital Ouagadougou….. “

*Amendment*

*“Studies will be conducted at two strategically chosen sites situated across the sub-Sahel, the geographical area where IPT in children is likely to be most effective. These are –*

*Boussé district, Burkina Faso. Boussé is a rural area situated 40 kilometers northwest of the capital Ouagadougou….”*

***Recruitment and randomization, page 8***

*“*After village meetings have been held in each study area to explain the nature of the trial, a census of children under five years of age resident in the study area will be conducted. At each site, approximately 2,100 children aged 3 – 59 months will be selected randomly from the census list for initial screening. The families of these children will be visited by a member of the field team and invited to participate in the study. If a family agrees that a child can join the trial, signed, informed consent will be obtained from a parent or guardian in the presence of an independent witness. Children will then be screened for their eligibility to join the trial until 2,000 have been entered. Screening will include administration of a questionnaire which asks about previous illnesses, clinical examination and measurement of height and weight”.

*Amendment*

*“After village meetings have been held in each study area to explain the nature of the trial, a census of children under five years of age resident in the study area will be conducted. At each site, approximately 3,100 children aged 3 – 59 months will be selected randomly from the census list for initial screening. The families of these children will be visited by a member of the field team and invited to participate in the study. If a family agrees that a child can join the trial, signed, informed consent will be obtained from a parent or guardian in the presence of an independent witness. Children will then be screened for their eligibility to join the trial until 3,000 have been entered. Screening will include administration of a questionnaire which asks about previous illnesses, clinical examination and measurement of height and weight”.*

Recruitment and randomization (last paragraph page 9)

Children will be individually randomized using a standard computer program which gives randomization within blocks of varying number. Separate randomization lists will be generated for each site. In order to reduce the chances of the investigators becoming aware of the drug code during the course of the trial, for example because of an increased incidence of vomiting in one group, eight randomization groups will be used, four for active drugs and four for placebo. Children enrolled in the trial will be issued with a photo identity card and the parents or guardian will be asked to present this whenever their child is taken to a health centre or hospital to ensure that the child is seen by a member of the study team.

“Children will be individually randomized using a standard computer program which gives randomization within blocks of varying number. Separate randomization lists will be generated for each site. In order to reduce the chances of the investigators becoming aware of the drug code during the course of the trial, for example because of an increased incidence of vomiting in one group, ten randomization groups will be used, five for active drugs and five for placebo. Children enrolled in the trial will be issued with a photo identity card and the parents or guardian will be asked to present this whenever their child is taken to a health centre or hospital to ensure that the child is seen by a member of the study team”.

**Sample size, last paragraph, page 10, paragraph 2**

“It is estimated that the rate of hospital admissions with malaria will be in the range of 20 to 60 per 1,000. Assuming a median incidence rate of 40 per 1,000, approximately 120 admissions with severe malaria might be anticipated in children in the control arm of the study. The study will, therefore, have at least 90% power to detect a 50% reduction in admission with severe malaria (from 40 to 20 per 1,000). The study will not be powered to detect an effect on mortality but deaths will be recorded and investigated for safety reasons”.

*Amendment*

*It is estimated that the rate of hospital admissions with malaria will be in the range of 20 to 60 per 1,000. Assuming a median incidence rate of 40 per 1,000, approximately 120 admissions with severe malaria might be anticipated in children in the control arm of the study. The study will, therefore, have at least 90% power to detect a 50% reduction in admission with severe malaria (from 40 to 20 per 1,000). Initially estimation of the impact of IPTc on admission with severe malaria was to be assessed using pooled data from three sites (MRTC, CNRFP and Navrongo Health Research Centre) and that the trial in Navrongo was suspended in June 2008 (due problems in getting ethical clearance), the number of children (2,000) who were to be enrolled at the Navrongo site has now been split between CNRFP and MRTC. Therefore the number of children to be enrolled in each of the two sites has been increased to 3,000 (1500 per arm). The study will not be powered to detect an effect on mortality but deaths will be recorded and investigated for safety reasons.*

**Intermittent preventive treatment (page 10 & 11)**

“Study children will be treated with a course of SP + AQ or matching placebos on three occasions during the malaria transmission season. Drugs will be pre-packaged to facilitate administration. Monthly doses will be timed to cover the peak transmission season at each site, July/August/September in Burkina Faso, July/Aug/Sept in Ghana and Aug/Sept/Oct in Mali. Doses of AQ and SP will be based on age as shown in Table 2.

**Table 2**. Dosage of study drugs.

Age in months Average Amodiaquine SP

weight (kg) tablets * tablets **

4- 6 6 1/4 1/2

*7*-11 9 1/2 3/4

12-35 13 3/4 1

36-59 18 1 1

Note. * Tablet given for 3 days;  ^** t^ablet given for 1 day

Each tablet of amodiaquine will contain 200 mg base and each tablet of SP 12.5 mg of pyrimethamine and 500 mg of sulfadoxine”

Drugs will be given under observation at a central point in the village. Children will be asked to stay at the distribution point for 30 minutes after drug administration. If vomiting occurs during this period, drugs will be re-administered. If vomiting recurs this will be noted but the drugs will not be given on a third occasion. Such children will not be excluded from the trial and they will be eligible to receive drugs at the subsequent monthly treatment. If a child misses the day set for treatment, a home visit will be made to enquire why the child was not brought for treatment and this will be recorded. If the family would like the child to continue with treatment but he or she was unable to attend on the specified day, then treatment will be reoffered for up to a maximum of 7 days after the designated date. Thus, no child will receive two doses of SP + AQ at an interval of less than three weeks”.

*Amendment*

“*Study children will be treated with a course of SP + AQ or matching placebos on three occasions during the malaria transmission season. Drugs will be pre-packaged to facilitate administration. Monthly doses will be timed to cover the peak transmission season at each site, July/August/September in Burkina Faso and Aug/Sept/Oct in Mali. Doses of AQ and SP will be based on weight as shown in Table 2. AQ will administered on the base of 10 mg/kg daily for 3 days and SP will administered as a single dose of 25mg/kg of sulfadoxine + 1.25 mg of pyrimethamine. Full tablets containing the exact daily dose (AQ) or the dose for a full course (SP) will be manufactured to avoid having to split tablets which may lead to administration of inadequate doses”.*

**Table 2**. Dosage of study drugs.

Weight groups (kg) Amodiaquine* SP**

Tablets (daily dose) Tablets (single dose)

Sulfadoxine Pyrimethamine

5-9 kg 70 mg 175 mg 8.75 mg

10-18 kg 140 mg 350 mg 17.5 mg

19 kg+ 220 mg 550 mg 26.25 mg

*Note. * Tablet given for 3 days;  ^**^ tablet given for 1 day*

*Drugs will be given under observation at a central point in the village. Children will be asked to stay at the distribution point for 30 minutes after drug administration. If vomiting occurs during this period, drugs will be re-administered. If vomiting recurs this will be noted but the drugs will not be given on a third occasion. Such children will not be excluded from the trial and they will be eligible to receive drugs at the subsequent monthly treatment. If a child misses the day set for treatment, a home visit will be made to enquire why the child was not brought for treatment and this will be recorded. If the family would like the child to continue with treatment but he or she was unable to attend on the specified day, then the first dose of treatment will be reoffered within 3 days and treatment must be completed within a maximum of 7 days after the designated date for each round. A dose missed on second of third day of treatment must be administered with 48 hours of the planned date. Thus, no child will receive two doses of SP + AQ at an interval of less than three weeks.*

***Safety of IPT with SP and AQ (page 11)***

“At the second and third treatment rounds, the families of each study child will be asked whether the child has had any symptoms that might have been drug related since the previous drug administration round. In addition, at each site, two hundred randomly selected children will be visited 7 – 10 days after the start of treatment and enquiries made about any symptoms that have occurred during the previous week”.

*Amendment*

*“Safety of SP and AQ will be monitored in all children on days 0, 1 and 2 of treatment administration, and on day 3 (day after the last dose of treatment for each treatment round). At the second and third treatment rounds, the families of each study child will be asked whether the child has had any symptoms that might have been drug related since the previous drug administration round”.*

**Surveillance of malaria morbidity (page 12 and 13)**

Page 12 (paragraph 1, starting line 3)

“…A finger prick blood sample will be obtained from all study children with fever (an axillary temperature of 37.5 ^0^ C or higher) or a history of fever within the previous 24 hrs for preparation of a blood film, collection of a filter paper sample measurement of hemoglobin (Hb) concentration and for a rapid diagnostic test (RDT) for malaria. Those who have a positive RDT for malaria will be treated immediately for malaria according to national guidelines (AS + AQ or AL). Children with anemia will be treated according to the national protocol”.

*Amendment*

“…*A finger prick blood sample will be obtained from all study children with fever (an axillary temperature of 37.5 ^0^ C or higher) or a history of fever within the previous 24 hrs for preparation of a blood film, measurement of hemoglobin (Hb) concentration and for a rapid diagnostic test (RDT) for malaria. Those who have a positive RDT for malaria will be treated immediately for malaria according to national guidelines (AS + AQ or AL). Children with anemia will be treated according to the national protocol*”.

Page 12 paragraph 3

“To obtain more information on the impact of IPT on the prevalence of malaria infection over time in the study population, at each site a random sample of 100 children will be visited at home once a week during the peak period of malaria transmission during the first and second years of the trial. During the intervening dry season, 100 children will be visited once every four weeks. The temperature of each child will be taken and a blood film obtained regardless of whether or not a child has fever. A rapid diagnostic test will be done on any child who has fever (axillary temperature of 37.5 ^0^ C or higher) or a history of fever within the previous 24 hrs and if this is positive treatment will be given according to national guidelines (AS+AQ or AL).

*Amendment*

*“To obtain more information on the impact of IPT on the prevalence of malaria infection over time in the study population, at each site a random sample of 150 children will be visited at home once a week during the peak period of malaria transmission during the first and second year of the trial. During the intervening dry season, 300 children will be visited once every four weeks. The temperature of each child will be taken and a blood film obtained regardless of whether or not a child has fever. A rapid diagnostic test will be done on any child who has fever (axillary temperature of 37.5 ^0^ C or higher) or a history of fever within the previous 24 hrs and if this is positive treatment will be given according to national guidelines (AS+AQ or AL)”.*

Page 12, paragraph 4

At the end of the malaria transmission season, a cross sectional survey will be undertaken at which each child will be examined, their height and weight recorded and a finger prick blood sample obtained for determination of Hb concentration, preparation of blood films and collection of a filter paper sample for subsequent studies on the prevalence of molecular markers of resistance”.

*Amendment*

*At the end of the malaria transmission season, a cross sectional survey will be undertaken at which each child will be examined, their height, arm circumference and weight recorded and a finger prick blood sample obtained for determination of Hb concentration, preparation of blood films and collection of a filter paper sample for subsequent studies on the prevalence of molecular markers of resistance”.*

**Surveillance for drug resistance (page 12 et 13)**

“Molecular markers associated with resistance to amodiaquine and to SP will be investigated in children with malaria parasitemia before and after the peak malaria transmission season to determine whether resistant parasites are selected by IPT. Samples will be collected as set out in Table 3.

**Table 3.** Samples for testing for molecular markers at each site.

Study group Number of samples Number likely to be Number for

collected positive for malaria molecular assays

Year 1

Random sample 500 200 100**

children < 5 years

before intervention *

Cross-sectional

survey – post intervention

IPT 1000 200 100**

Placebo 1000 500 100**

Year 2

Cross-sectional

survey

IPT 1000 500 100**

Placebo 1000 500 100**

Notes.

* These children will not be included in the trial but will come from the same area.

** Random sample from the positive blood samples available.

Thus, during the first year of the study approximately 300 samples will be tested from each site and during the second year 200 giving a total of 1,500 over the two years of the trial”.

*Amendment*

*“Molecular markers associated with resistance to amodiaquine and to SP will be investigated in children with malaria parasitemia before and after the peak malaria transmission season to determine whether resistant parasites are selected by IPT. At baseline (before treatment administration) children will be sampled from a census database list for the prevalence of markers of resistance to SP and AQ. These children will have a clinical examination, a thick and thin blood film and filter paper blood spot prepared, and haemoglobin concentration will be determined. Samples will be collected as set out in Table 3.*

***Table 3.*** *Samples for testing for molecular markers at each site.*

*Study group Number of samples Number likely to be Number for*

*collected positive for malaria molecular assays*

*Year 1*

*Random sample 750 300 150***

*children < 5 years*

*before intervention **

*Cross-sectional*

*survey – post intervention*

*IPT 1500 300 150***

*Placebo 1500 750 150***

*Year 2*

*Cross-sectional*

*survey*

*IPT 1500 750 150***

*Placebo 1500 750 150***

*Notes.*

** These children will be from the study village, but will not be included in the trial.*

*** Random sample from the positive blood samples available.*

*Thus, during the first year of the study approximately 450 samples will be tested from each site and during the second year 300 giving a total of 1,500 over the two years of the trial”.*

***Laboratory methods, page 14***

“Thick blood films will be air dried, stained with Giemsa and examined for malaria parasites by two well-trained technicians. One hundred high power fields will be counted before a film is declared negative”.

*Amendment*

*“Thick blood films will be air dried, stained with Giemsa and examined for malaria parasites by two well-trained technicians. A slide must be fully examined (all fields) before a film is declared negative”*.

1. If you fall within one of the first five categories please include your IRS tax determination letter in Appendix A. If you are a non-U.S. charitable organization, please see fiscal status link [↑](#footnote-ref-2)
